# Passive Transport across Cell Membranes beyond the Overton Rule: Insights from Solute Exchange in Vesicles and Molecular Dynamics of Atropisomers

**DOI:** 10.1021/acsami.4c22459

**Published:** 2025-04-10

**Authors:** Margarida M. Cordeiro, Alexandre C. Oliveira, Paulo E. Abreu, Luis G. Arnaut, Maria João Moreno, Luís M. S. Loura

**Affiliations:** † Coimbra Chemistry Center, Institute of Molecular Sciences (CQC-IMS), University of Coimbra, 3004-535 Coimbra, Portugal; ‡ Department of Chemistry, University of Coimbra, 3004-535 Coimbra, Portugal; § Center for Neuroscience and Cell Biology (CNC), University of Coimbra, 3004-535 Coimbra, Portugal; ∥ Faculty of Pharmacy, University of Coimbra, 3000-548 Coimbra, Portugal

**Keywords:** drug bioavailability, 3D molecular descriptors, partition diffusion, membrane permeability

## Abstract

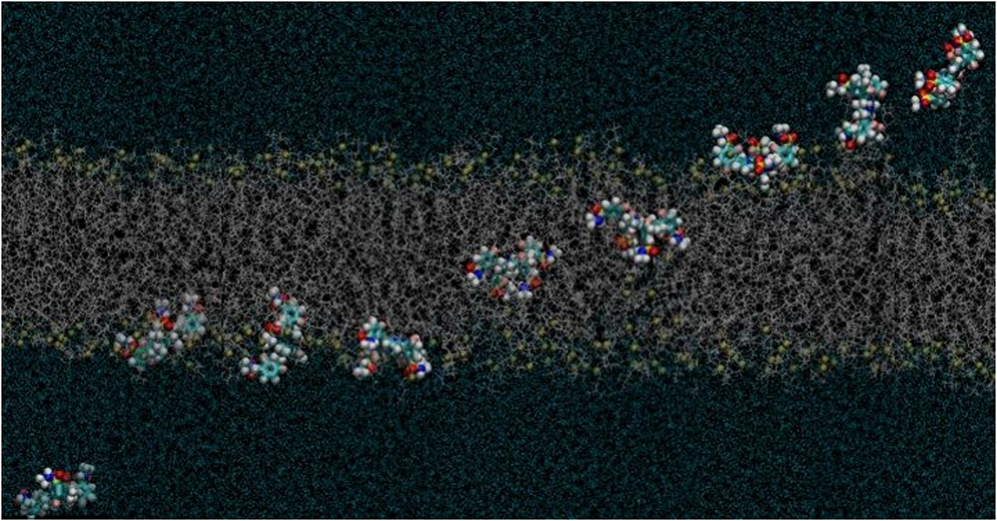

Bioavailability of a drug is critically dependent on its cell membrane permeability. Empirical rules guiding drug design consolidated the dogma that large molecules cannot cross cell membranes by passive diffusion. However, the more amphiphilic atropisomers of redaporfin, an 1135 Da bacteriochlorin photosensitizer used in photodynamic therapy, exhibited fast cell uptake and high photodynamic activity *in vitro*. This motivated detailed studies of redaporfin atropisomers and their interactions with cell membrane models. Experimental studies on membrane affinity, permeation rates, and exchange dynamics were complemented by molecular dynamics simulations, to reveal the nature of the interactions between the atropisomers and lipid bilayers, the orientation and location of the membrane-bound atropisomers, free energy profiles, and mechanisms governing membrane permeation. Our results indicate that the asymmetric distribution of the *meso*-phenyl sulfonamide groups (atropisomer α_4_) generates a large amphiphilic moment. This enhances its membrane affinity and positions the bacteriochlorin ring deeper in the membrane. However, these strong membrane interactions result in a slow exchange of α_4_ between lipid membranes, restricting its distribution in complex, membrane-rich environments. In contrast, the more symmetrical atropisomer αβαβ exhibits approximately 10-fold lower membrane affinity and localizes closer to the membrane–water interface. This weaker interaction facilitates rapid exchange between membranes, occurring within minutes at 37 °C. Molecular dynamics simulations reveal relatively low energy barriers for membrane translocation, consistent with experimentally estimated fast translocation. Distinct permeation mechanisms were observed for the two atropisomers, providing insights into their differential behavior in passive membrane transport. In particular, the fast cell uptake of the α_4_ atropisomer is properly described by the bind-flip mechanism, where the sulfonamide groups first approach the bilayer in a “binding” mode, and then the molecule “flips” to place the macrocycle in a more internal position. Our results show how amphiphilicity and conformation flexibility are critical determinants in the cellular internalization of large molecules.

## Introduction

Efficient delivery of macromolecules to intracellular targets has the potential to dramatically expand therapeutic options for devastating diseases, but remains a longstanding challenge.^1^ Macromolecules, broadly defined as entities with a molecular weight (MW) larger than 1 kDa and a Stokes–Einstein radius larger than 1 nm,^2^ are usually uptaken by cells through endocytosis-dependent routes that often result in <10% cytosolic release of the delivery cargo.^3^ It came as a surprise that redaporfin (MW = 1135 Da), a porphyrin derivative in clinical trials for photodynamic therapy (PDT)^4,5^ of advanced head and neck cancer,^6^ has four separable atropisomers that cross cell membranes by passive diffusion with widely different rates. One of the atropisomers (named α_4_ because all bulky substituents are on the same side of the macrocycle) is internalized by the cells much faster than the other ones, while another atropisomer (named αβαβ because the bulky substituents alternate in the sides of the macrocycle) is very slowly internalized. A similar phenomenon was subsequently found for strapped porphyrins.^7^ Although atropisomers differ only on the rotation of a carbon–carbon single bond,^8^ this minor structural difference is responsible for differences in passive cell internalization that change *in vitro* phototoxicity from dramatic to negligible at the same drug and light doses.^9^

It is widely recognized that kinetics of drug–membrane interactions plays a major role in drug bioavailability and pharmacokinetics and overall in its bioactivity. This was enshrined one century ago by the Overton Rule that in modern language states that the entry of a small molecule into a cell is proportional to its solubility in the cell’s boundary.^10^ Hence, the cell permeability of a drug should be proportional to its partition coefficient from water to organic phases, the latter mimicking the lipid bilayer of cell membranes. Although this rule remains widely accepted today for passive transport of drugs, i.e., when the drug does not have specific carriers (usually proteins) for their transport, it adds incongruity to the different cell uptake of atropisomers α_4_ and αβαβ because their *n*-octanol:water partition coefficients are very similar: log *P*_OW_^α4^ = 2.9 and log *P*_OW_^αβαβ^ = 2.6, respectively.^9^

This work reports experimental and computation efforts to understand passive diffusion of large molecules across the lipid bilayers and how they can evade the Overton rule. The experimental work makes use of lipid vesicles (liposomes), which are especially relevant to model passive transport through the plasma membrane. We use large unilamellar vesicles (LUVs) and the strong intermolecular distance dependence of Förster resonance energy transfer (FRET)^11^ to interrogate atropisomer localization and exchange kinetics in phospholipid bilayers. This is complemented by molecular dynamics (MD) simulations to obtain a molecular understanding of passive transport through cell membranes.^12^ MD simulations provide insight into the transition state of membrane translocation and, consequently, into the mechanism of passive transport. We show that these tools support a mechanism characterized by a first step where the approach of the α_4_ atropisomer to the bilayer is accompanied by the orientation of its polar groups toward the lipid headgroups, a second step where the α_4_ atropisomer “flips” around this location to reach a more internal position in the bilayer while maintaining its polar groups at the interface, and a third step in the interior of the bilayer where the atropisomer flips again to orient the polar groups toward the opposite interface. This bind-flip mechanism should be of relevance for amphiphilic macromolecules and may drive the design of macromolecules with a greater potential to attain intracellular targets.

## Results and Discussion

### Atropisomers Molecular Structure

**Figure 1:**
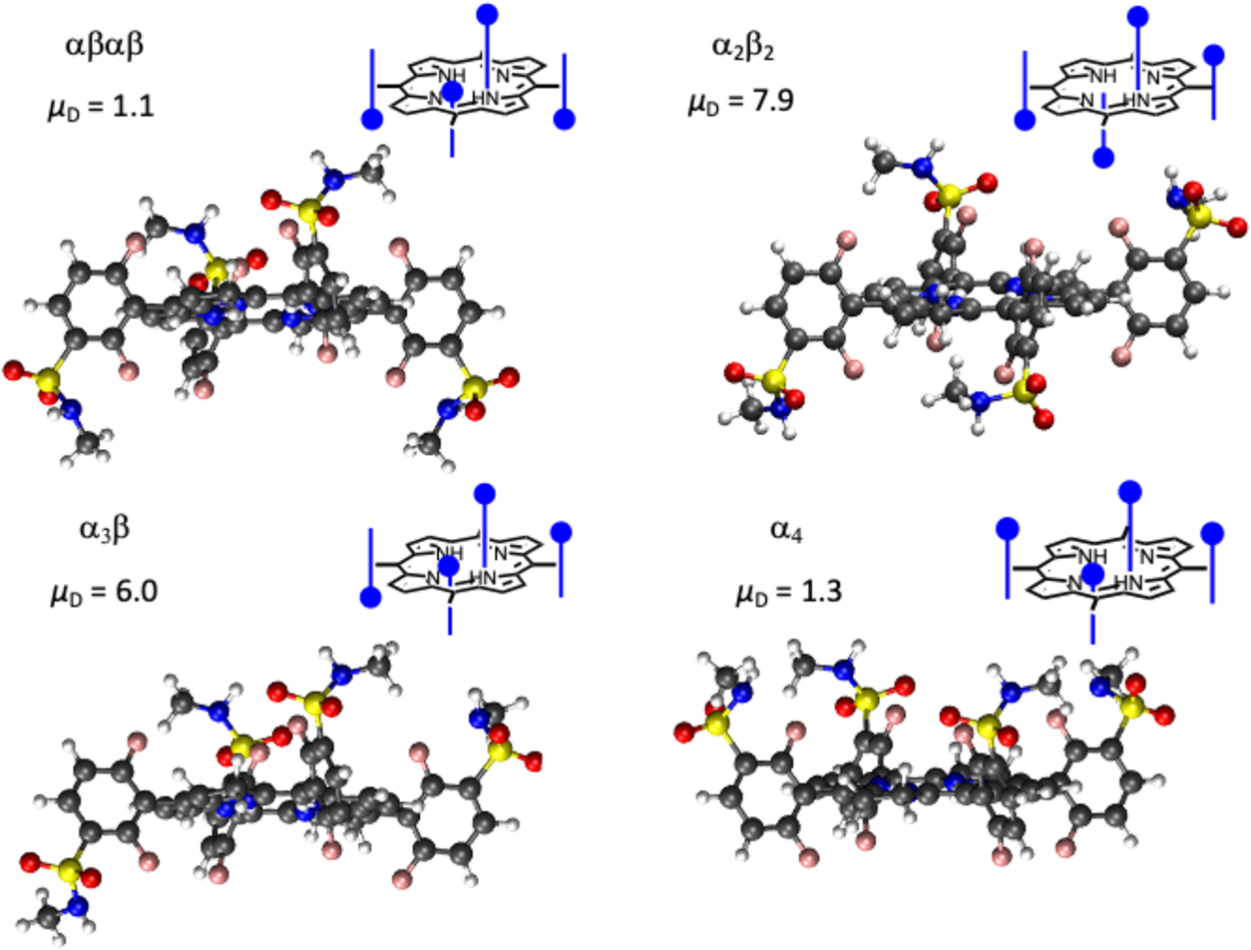
Molecular structures of the four atropisomers of redaporfin. Color code for the atoms: dark gray, carbon; white, hydrogen; yellow, sulfur; dark blue, nitrogen; light pink, fluorine.

Electronic structure calculations made at the B3LYP/G-31G(d,p) level of theory after geometry optimization at the same level using GAMESS-US,^13^ offer a detailed picture of the molecular structures and dipole moments of the four separable atropisomers of redaporfin (Figure 1). The atropisomer with the four polar groups on the same side of the macrocycle (α_4_) is not the atropisomer with the highest dipole moment in the most stable conformation in the vacuum. In fact, its dipole moment is not very different from that of the atropisomer with the polar groups in alternate sides of the macrocycle (αβαβ). Atropisomers α_2_β_2_ and α_3_β both have much higher dipole moments than those of α_4_ and αβαβ. Charge distributions shown in Figure S1 help us to understand that the local dipole moments of the S=O bonds tend to offset each other. Atropisomers α_4_ and αβαβ have the most distinct biological behavior,^9^ and subsequent calculations and experiments focused on these two atropisomers to obtain a detailed picture of their interaction with phospholipid bilayers.

### Partition of Redaporfin to LUVs

**Figure 2:**
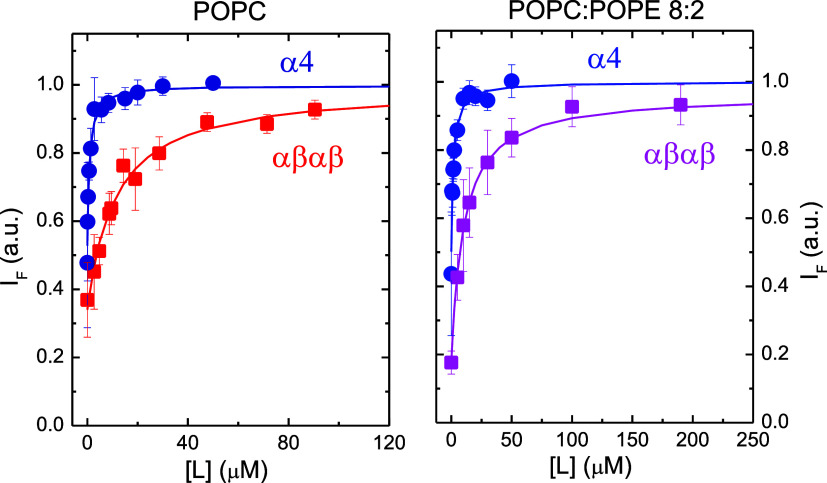
Variation of redaporfin fluorescence intensity (λ_exc_/λ_em_ = 508/752 nm) at 37 °C due to partition to lipid bilayers of POPC or POPC:POPE (4:1 molar ratio); note the different abscissae scale in the two plots. The final concentration of the redaporfin atropisomers was 2.1 nM, added from stock solutions in DMSO leading to 5 vol % DMSO. The average and standard deviation of 4 independent experiments are shown (α_4_: blue solid circle and light blue solid circle; αβαβ, red box solid and pink box solid). The lines correspond to the best fit of eq 1, leading to a partition coefficient to the POPC membranes *K*_P_^POPC^= 1.5 × 10^6^ for α_4_, and 1.1 × 10^5^ for αβαβ; and to the POPC:POPE membranes *K*_P_^PC:PE^ = 7.2 × 10^5^ for α_4_, and 1.2 × 10^5^ for αβαβ.

Typical results obtained for changes in redaporfin fluorescence due to partition to LUVs composed of POPC are shown in Figure 2. The conditions used result from an extensive optimization of redaporfin and lipid concentrations, incubation time, method of redaporfin addition, and final DMSO concentration (Section S2). At the very low redaporfin concentration employed (2 nM), the dependence of the redaporfin fluorescence intensity with the lipid concentration is well described by a simple partition, eq 1, leading to log *K*_P_^POPC^ = 6.2 ± 0.2 and 5.0 ± 0.1, for α_4_ and αβαβ, respectively (at 5 vol % DMSO and 37 °C, *p*-value <10^–5^ between the two atropisomers). The partition for both redaporfin atropisomers to LUVs is several orders of magnitude higher than their octanol/water partition coefficients (log *P*_OW_ = 2.9 and 2.6 for α_4_ and αβαβ, respectively^9^). Obviously, log *K*_P_^POPC^ does not measure the same properties as log *P*_OW_. A notable difference between POPC vesicles in water and the classical phase separation between *n*-octanol and water is the much higher interfacial area of the vesicles. Amphiphilic molecules that are stabilized at polar/apolar interfaces will partition better to POPC LUVs than to *n*-octanol in water. The difference between log *K*_P_^POPC^ and log *P*_OW_ reflects the amphiphilicity of αβαβ and, especially, that of α_4_, whereas the magnitude of *P*_OW_ measures the balance of hydrophobicity/hydrophilicity in the bulk of the solvents.

Redaporfin partition to membranes containing POPC and POPE (4:1 mol) was characterized to evaluate specific interactions with lipids. The lipid POPE was selected due to its high abundance in biomembranes,^14,15^ and possible establishment of hydrogen bonds from the ethanolamine groups (donors) to redaporfin sulfonamide substituents (acceptors). Distinct effects of POPE are therefore expected for the membrane affinity of these atropisomers if hydrogen bonding is important for their interactions. Data in Figure 2 shows a decrease in the affinity of α_4_ for the membrane (log *K*_P_^PC:PE^ = 5.9 ± 0.2, *p*-value <10^–3^ relative to log *K*_P_^PC^), while that of αβαβ remains essentially unchanged (log *K*_P_^PC:PE^ = 5.1 ± 0.2). The decrease in the solvation ability of the lipid bilayer with POPE observed for α_4_ suggests that hydrogen bonding with the ethanolamine group does not contribute significantly to the membrane affinity of this atropisomer. The gel-to-fluid transition of POPE (*T*_m_ = 24 °C) is higher than that of POPC (*T*_m_ < 0 °C),^16^ and the decrease observed in α_4_ affinity may be due to a decrease in membrane fluidity. This may impair the penetration of α_4_ in the bilayer nonpolar region, thus decreasing the interactions established with the membrane.

The redaporfin-to-lipid molar ratios can be calculated from the partition coefficients in Figure 2. For 2 nM redaporfin at half-partition to the LUVs, this ratio is ∼1000 for α_4_ and ∼10,000 for αβαβ, and remains >500 for all lipid concentrations employed. When the concentration of redaporfin is increased to 50 nM, the partition coefficient of α_4_ decreases to ∼25% of the value at 2 nM, which corresponds to a lipid-to-redaporfin ratio of ∼500 at half-partition. These high ratios reveal that the local concentration of redaporfin in the membrane is always very low and, consequently, that the decrease in K_P_ is not due to saturation of redaporfin in the membrane.^17,18^ Alternatively, the increase in concentration may lead to the aggregation of redaporfin in the aqueous phase. In fact, at 25 nM, αβαβ shows a strong decrease in the fluorescence intensity over time when added to the aqueous medium or in the presence of low lipid concentrations (Figure S2). This effect leads to a decrease in the apparent partition coefficient and a poor fit of the equation assuming simple partition, with the fluorescence dependence on lipid concentration following a sigmoidal shape even for low incubation times. Deviations from the behavior expected for simple partition become more accentuated as the concentration of redaporfin increases (Figure S3). This shows that the presence of the additional equilibrium of solute aggregation in one of the phases competes with the partition and leads to lower apparent partition coefficients. In agreement with this effect, a strong decrease is observed in the apparent partition coefficient to POPC LUVs obtained at a total redaporfin concentration of 10 μM.^19^

### Unrestrained MD Simulations of Redaporfin Interaction with Bilayers

**Figure 3:**
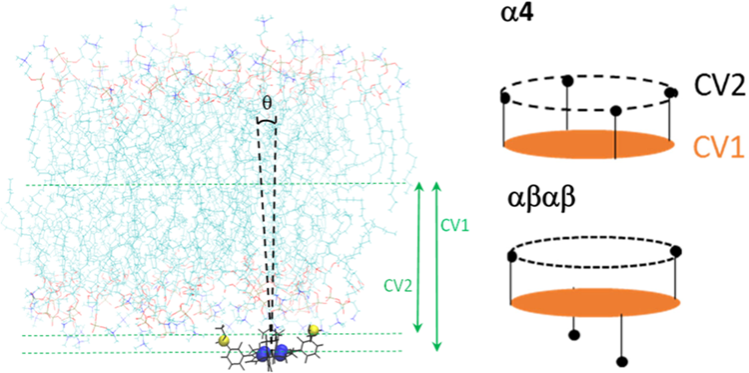
Left: illustration of the definition of the collective variables CV1 and CV2 for an α_4_ molecule near a bilayer and of the angle between the directions normal to the bacteriochlorin ring system and the bilayer plane, θ. The macrocycle nitrogen atoms (center of mass used to define CV1) and two opposite sulfur atoms (center of mass was used for defining CV2) are highlighted in blue and yellow, respectively. Right: illustration of how the definition of the CVs relates to α_4_ and αβαβ atropisomers. All *meso*-phenyl sulfonamide substituents (and therefore all S atoms) are on the same side of the tetrapyrrole macrocycle for α_4_. In contrast, for αβαβ, the two pairs of diametrically opposite S atoms (one of which was used for defining CV2) are on different sides of the macrocycle.

Three independent systems composed of two α_4_ or αβαβ molecules near the interface of a hydrated 104 POPC:26 POPE bilayer were simulated for 1 μs each, as described in the Materials and Methods section. Figure 3 shows the collective variables (CV1 and CV2) defined to describe the position and orientation of the molecules relative to the bilayer. Figure S4 shows the time evolution of the CVs of each simulated molecule in the three replicates of the two systems. It is clear that all molecules move toward their equilibrium locations early (first 100–200 ns) during the simulations. In all runs but one, the two molecules ended up inserted into opposite membrane leaflets. The sole exception, the run depicted in the bottom right panel of Figure S4, occurred for one αβαβ replicate. It should be noted that this has no bearing on the trajectory analysis, as no aggregation was apparent for the molecules in the same leaflet, and both location and orientation of these two molecules were within the ranges obtained for the other simulations of the same system.

**Figure 4:**
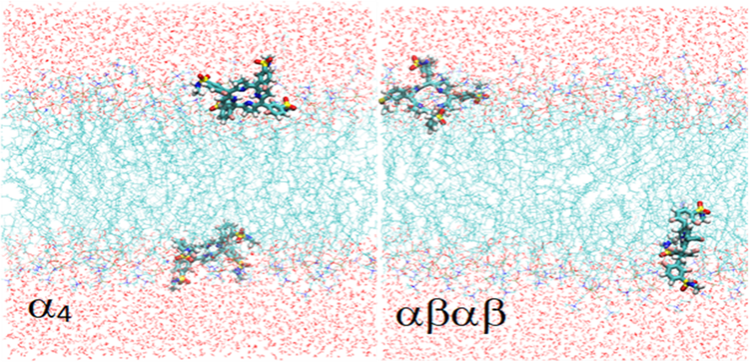
Typical configurations of the atropisomers after equilibration in the system.

Figure 4 illustrates the representative orientations of the atropisomers after equilibration. The bacteriochlorin ring of the α_4_ atropisomer is deeper in the bilayer, and its polar substituents are pointed outside, toward the aqueous media of both sides of the bilayer. The αβαβ atropisomer is partially inserted in the bilayer, in a balancing act of hydrophobic interactions of its nonpolar groups inside the bilayer and hydrophilic interactions at the interface and with bulk water. The study of the average locations of the atropisomers in the system, at equilibration, allows for a more quantitative description of the interactions.

**Figure 5:**
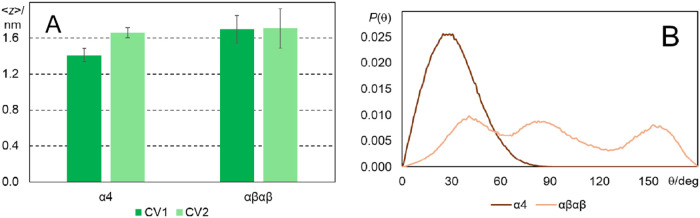
(A) Average transverse locations of CV1 and CV2. (B) Angular probability density function of the tilt of the CV1 → CV2 vector relative to the bilayer normal for the two atropisomers.

Figure 5A shows the average transverse locations obtained for the two CVs of both atropisomers. These values lie all slightly inside the average location of the phosphorus atoms (1.88 nm). CV1 and CV2 of αβαβ have identical average locations (1.70 ± 0.15 and 1.71 ± 0.22 nm, respectively). Although CV2 of α_4_ points to a location similar to that of αβαβ (1.66 ± 0.06 nm), CV1 points to a clearly deeper location (1.41 ± 0.07 nm). This means that the macrocycle of α_4_ inserts at the level of the ester groups of the phospholipids, clearly deeper than that of αβαβ and deeper than the sulfonamide groups of both isomers, which on average reside in the headgroup region. Overall, these results are in good agreement with those experimentally estimated for the redaporfin atropisomer mixture, 1.04 nm from the center of the bilayer, using parallax fluorescence quenching.^19^ Binning of the transverse locations *z* of CV1 and CV2 allows calculation of the relative frequency of a given location, *d*(*z*), from which an estimate of the partial free energy profile along the *z* coordinate can be obtained (Figure S5). From these curves, it is already apparent that a barrier exists for translocation with a height of at least 25 kJ mol^–1^, consistent with the absence of redaporfin molecules moving across to the opposite leaflet during these unrestrained simulations.

The locations |CV1| < |CV2| indicate that α_4_ displays a clear orientation in the bilayer, with the substituents pointing toward the water medium and the macrocycle residing at a more internal position. However, the locations CV1 ≈ CV2 of αβαβ cannot distinguish between a random orientation within the bilayer or an orientation of the macrocycle perpendicular to the bilayer plane, since both possibilities lead to similar average locations of CV1 and CV2 for this molecule.

Further information about locations can be extracted from distributions of the tilt angle θ of the CV1 → CV2 vector relative to the bilayer normal (see Figure 3 for illustration). Low θ values denote a perpendicular orientation of this vector relative to the bilayer plane (pointing to the aqueous medium), θ ≈ 90° corresponds to alignment of the vector with the bilayer plane (perpendicular orientation of the bilayer and macrocycle planes), and obtuse θ indicate CV1 → CV2 pointing toward the center of the bilayer. Figure 5B shows that the distribution of α_4_ is centered around low θ values and spans only acute angles. Accordingly, the mean value over the time averages of the six simulated molecules of this atropisomer is 31 ± 4°. A trimodal distribution was obtained for αβαβ, with peaks in both the acute and obtuse angle region (expected from the symmetry of the molecule) and an additional one at ca. 80–90°, resulting from conformations where the bacteriochlorin and the bilayer are in perpendicular planes (αβαβ molecule in the lower bilayer leaflet in the right panel of Figure 4). It is possible that simulation of more molecules would lead to a broader, less structured distribution, but analysis of the angle distribution for each molecule shows that the orientation is maintained throughout the simulation, indicating a high energy barrier between distinct stable orientations (Figure S6). In any case, we can infer that membrane-inserted α_4_ has one single most stable orientation, while αβαβ has several possible orientations with similar energy.

Instant dipole moment values were calculated for the six simulated molecules of each atropisomer (Figure S7). Because of the conformational flexibility arising from the *meso*-phenyl sulfonamide substituents attached to the bacteriochlorin macrocycle, a wide range of values are displayed throughout the simulations. Compared to those obtained for the optimized structure in a vacuum (Figure 1), the average values recorded for both atropisomers are considerably higher, viz. 15.6 D (standard deviation 0.8 D, *n* = 6) and 9.5 D (standard deviation 1.2 D, *n* = 6) for α_4_ and αβαβ, respectively. While minimal values of essentially zero are observed for αβαβ, the dipole moment of α_4_ was never lower than 1.6 D for any of the six simulated molecules. On the other end, maximal values of 29.5 D (α_4_) and 24.1 D (αβαβ) were observed. Overall, α_4_ and αβαβ have similar dipole moments in the vacuum calculated at the B3LYP/6-31G(d,p) level of theory (Figure 1), but in the MD simulations performed with the phospholipid bilayers the dipole of α_4_ increases much more than that of αβαβ. Using MD simulations, it is common to find higher dipole moments of polar molecules in polar solvents than in the vacuum.^20^ The difference may be partially due to the different levels of theory used in geometry optimization and calculation of the electronic structure of the molecules,^20^ and to changes in the environment.^21,22^ However, the dipole moment increases from 1.3 D in the vacuum to 15.6 ± 0.8 D in the bilayer for α_4_ and from 1.1 to 9.5 ± 1.2 D for αβαβ, far exceeding the range of values found in the literature. This can be understood considering that the polar environment stabilizes conformations with higher dipole moments that are not energetically accessible in the vacuum. Indeed, using the geometries optimized in the vacuum for α_4_ and αβαβ, and the charges used for MD simulations, results in dipole moments of 6.54 and 6.40 D, respectively. The additional increase to 15.6 ± 0.8 or 9.5 ± 1.2 D, can be assigned to contributions from conformations of high dipole moment that have low energies in polar environments. A detailed analysis of the dependence of the overall dipole moment with the depth location and orientation of the redaporfin molecules in the lipid bilayer (Figures S8–S10) shows that the dipole moment of αβαβ is mostly aligned with the bacteriochlorin ring and at ≈20° from the ring normal in the case of α_4_. The results point toward a significant contribution of the interaction between redaporfin dipole moment and the lipid bilayer dipole potential, stabilizing the association of αβαβ perpendicular to the membrane surface, and partially justifying the smaller decrease observed for the affinity of this atropisomer for the membrane with the higher dipole potential (PC:PE 8:2).^23^

**Figure 6:**
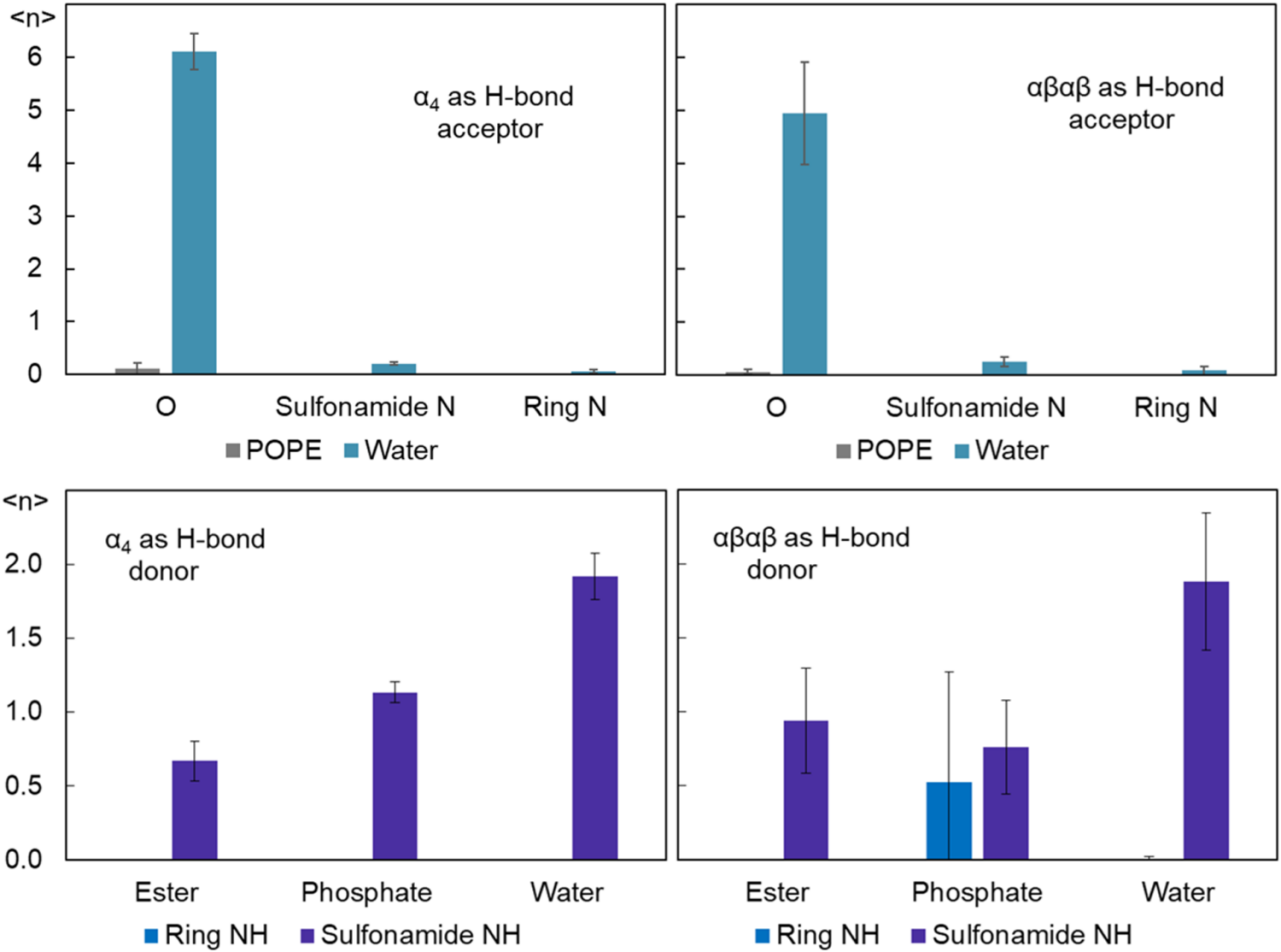
Average number of H bonds, ⟨*n*⟩, per redaporfin molecule. Top panels: redaporfin acting as H-bond acceptor from water (each column concerns different accepting redaporfin atoms) or POPE. Bottom panels: Redaporfin sulfonamide groups or NH in the macrocycle ring donating H bonds to water or lipid groups. Left panels refer to α_4_, whereas the right panels show data from αβαβ.

Redaporfin has both H-bonding donor and acceptor groups, able to form H bonds with surrounding phospholipid or water molecules, which may affect their location and dynamics within the lipid bilayer. These interactions were identified from geometric criteria (hydrogen-donor–acceptor angle <30°, donor–acceptor distance <0.35 nm) during the simulation, and averaged over the simulation time for the six simulated molecules of each atropisomer, to obtain averages of the instant number of H bonds per redaporfin molecule. Figure 6 shows that for the most part, the two atropisomers have similar behaviors. For example, H bonds to water are consistently found, both from water OH to accepting (mainly sulfonamide oxygen; Figure 6, top panels) atoms and from sulfonamide NH groups to water atoms (Figure 6, bottom panels). A nonsignificantly higher number of hydrogen bonds donated by water was observed for α_4_, possibly reflecting that, at any given time, some sulfonamide oxygen atoms of αβαβ are facing the bilayer interior or inserted in the bilayer and thus are less accessible to accept H bonds. The differences in the location and orientation of the sulfonamide groups do not considerably affect their H-bonding to the solvent.

While redaporfin N and O atoms could conceivably act as acceptors from the amine group of POPE, this interaction is seldom found for both atropisomers (Figure 6, top panels). At variance, redaporfin sulfonamide NH groups are frequently involved as H-bond donors to phosphate or ester lipid groups (Figure 6, bottom panels). While αβαβ displays a higher average number of H bonds to ester groups, α_4_ makes more H bonds to phosphate groups. This is consistent with the view that αβαβ has some of its substituents in a more inward orientation, less prone to H-bonding to headgroup lipid groups, but more favorable to bonding to the carbonyl/ester O atoms. Regarding H bonds from ring NH groups, these are only observed in αβαβ. The probable reasons for this are the more external average location of the ring N atoms in αβαβ compared to α_4_, and the existence of perpendicular ring orientations in αβαβ, meaning that one of the opposite NH groups may have a particularly external location. Together, these observations imply that some αβαβ-ring NH donors may be (at least occasionally) able to act as H donors to phosphate acceptor atoms. The large variation in the average frequency reflects the different possible orientations of the macrocycle ring of αβαβ, of which only that perpendicular to the bilayer plane is compatible with H bonding. At variance, the depth and orientation of the macrocyle ring of α_4_ precludes H bonding from the ring NH groups in this isomer.

The possibility of local membrane perturbation by redaporfin was also assessed from inspection of the acyl chain order parameters, −*S*_CD_. Order parameter profiles were calculated for phospholipids located at different distances *R* to the redaporfin molecule inserted in the same leaflet (Figure S11). Both atropisomers induce slight local (*R* < 0.6) ordering of the first segments of the *sn*-1 acyl chain (up to the fifth or sixth C atom in α_4_ and αβαβ, respectively). For lower acyl chain carbon atoms, a disordering effect is observed, most pronouncedly in α_4_. This atropisomer induces a larger degree of local perturbation on account of its macrocycle orientation mostly aligned with the bilayer plane. This leaves a void below the ring, which is filled with chains from neighboring lipids. In turn, these chains become less aligned with the bilayer normal, resulting in a lower order parameter. This effect is less noticeable in αβαβ. For this atropisomer, two out of six αβαβ molecules kept their bacteriochlorin ring mostly aligned with the membrane plane normal during the simulations (Figure S6, right), while the other four had orientations closer to that preferred by α_4_. Insertion of a rigid ring mostly perpendicular to the membrane plane is more prone to order nearby lipid acyl chains and hence the overall diminished disordering effect induced by αβαβ. For both atropisomers, effects become negligible for *R* > 1.0 nm.

### Kinetics of Redaporfin Interaction with LUVs

The characterization of the structure and equilibria of redaporfin atropisomers in phospholipid bilayers must be complemented by the study of their kinetics to provide a proper understanding of the processes underlying the differential biological activity of these atropisomers. We investigated the rates of α_4_ and αβαβ exchange in POPC:POPE lipid vesicles preparing these LUV with 1 mol % NBD-DPPE and 0.5 mol % redaporfin. NBD-DPPE is a fluorescence probe where the dipalmitoyl-*sn*-glycero-3-phosphoethanolamine (DPPE) lipid is labeled with the 7-nitro-2,1,3-benzoxadiazol-4-yl (NBD) in the ethanolamine group (λ_exc_/λ_em_ = 460/530 nm), and is commonly used to study model membranes because of its relatively high fluorescence quantum yield Φ_F_ = 0.32.^24^ There is a good overlap between the fluorescence of this probe and the absorption of redaporfin, which has ε_505_ = 7.0 × 10^4^ M^–1^ cm^–1^ (Figure S12).^25^ Using these values, the experimental redaporfin absorption spectra and NBD-DPPE emission spectra, the refractive index of methanol,^26^ and the equations in Section S4, we calculate a Förster Radius comparable to the thickness of the lipid bilayer (*R*_0_ = 4.1 nm), which means that efficient energy transfer will take place from NBD-DPPE to redaporfin when they are located in any of the two membrane leaflets.

**Figure 7:**
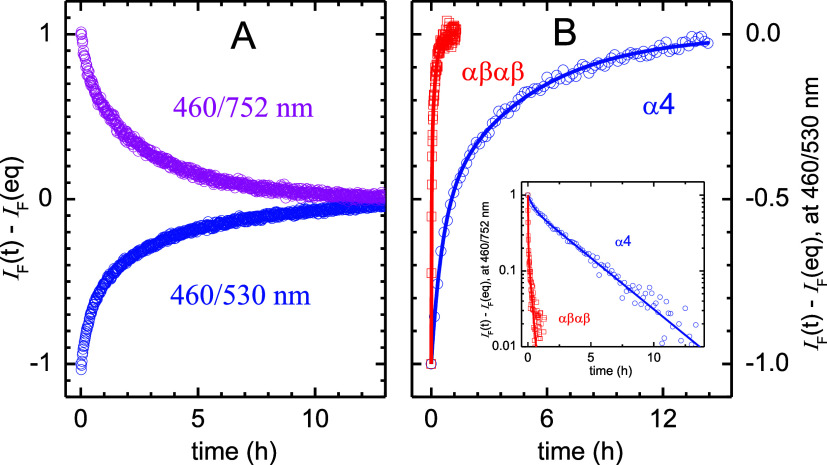
Rate of exchange of α_4_ between POPC:POPE 4:1 LUVs at 37 °C. (A) Increase in the fluorescence of NBD-DPPE (λ_ex_/λ_em_ = 460/530 nm, blue circle) and decrease in the fluorescence of α_4_ after energy transfer from NBD-DPPE (λ_ex_/λ_em_ = 460/752 nm, Pink circle), when donor LUVs containing 1 mol % NBD-DPPE and 0.5 mol % α_4_ are mixed with blank LUVs. (B) Comparison of the variation of the fluorescence intensity due to exchange of α_4_ (blue circle, blue long dash) or αβαβ (red square, red long dash), when followed from the increase in the fluorescence of NBD-DPPE. The lines are the best fit of biexponential functions, the average parameters are given in Table 1. The inset shows the corresponding decrease in the fluorescence of redaporfin when NBD-DPPE is excited at 460 nm (note the logarithmic ordinate scale).

Redaporfin exchange was followed through the time-dependent decrease in the FRET efficiency when blank LUVs are added. Typical results obtained for the increase in the fluorescence intensity of the donor (NBD-DPPE) and the decrease in the fluorescence intensity of the α_4_ atropisomer are shown in Figure 7A. A very slow exchange is observed, with the variation in the fluorescence intensity occurring over several hours. The kinetic profile is essentially equivalent when followed through variations in the donor (NBD-DPPE) or in the acceptor (α_4_) fluorescence, confirming that it is due to the decrease in FRET efficiency.

The rate of NBD-diCnPE transfer in POPC LUVs has been characterized previously for the case of acyl chains with *n* = 6–14 carbons,^27^ and from the trend observed a rate constant close to 1 × 10^–7^ s^–1^ was calculated (τ ≅ 3 months). Therefore, the variation in fluorescence observed on the scale of hours must reflect the exchange of the α_4_ atropisomer. Nevertheless, given the distinct lipid composition and the very slow exchange observed, control experiments were performed to confirm that the variations in the fluorescence intensity are due to the exchange of α_4_ and not of NBD-DPPE. The time dependence of NBD-DPPE fluorescence with the addition of blank LUVs, presented in Section S5, proves that the variations in the FRET efficiency observed in Figure 7A are exclusively due to α_4_ exchange.

The rate of exchange of the αβαβ atropisomer was also characterized in Figure 7B. The rates of exchange obtained by fittings to the data in Figure 7 and reported in Table 1 show that the exchange of αβαβ at 37 °C is 2 orders of magnitude faster than that of α_4_. A faster exchange of αβαβ could be anticipated given its lower affinity to the POPC:POPE membrane, . However, this lower membrane affinity cannot explain the 2 orders of magnitude increase in the exchange rate.

**Table 1:** Average Values and Standard Deviation^a^ for the Partition Coefficients and Parameters for the Exchange of Redaporfin Atropisomers in POPC:POPE 4:1 LUVs at 37 °C

	parameter	α_4_	αβαβ
partition	log *K*_P_	5.9 ± 0.2	5.1 ± 0.2
exchange^b^	*k* (s^–1^)	1.5 × 10^–4^	1.9 × 10^–2^
	*E*_a_ (kJ/mol)	125 ± 8	121 ± 4
	*A* (s^–1^)	2.6 × 10^17^	5.6 × 10^18^

a The average and standard deviation were calculated from log *K*_P_ and log *k*, as these are the parameters with a normal distribution; from these, the average values of the parameters were calculated.

b Includes only the results from the increase in the fluorescence intensity of the FRET donor (NBD-DPPE), corresponding to 25 replicates for α_4_ and 10 for αβαβ at 37 °C.

To provide further insights regarding the reasons for the slower kinetics of α_4_, the effect of temperature on the rate of exchange was characterized. The rate of exchange of α_4_ was followed in a conventional fluorimeter through the variation in the fluorescence intensity of NBD-DPPE (at λ_ex_/λ_em_ = 460/530 nm), and that of α_4_ due to the decrease in FRET from NBD-DPPE (at λ_ex_/λ_em_ = 460/750 nm), both leading to similar results. Variations at both wavelengths were also followed for αβαβ at 25 °C, leading to similar results, as well. The exchange of αβαβ at higher temperatures was, however, too fast to be followed in the conventional fluorimeter and had to be characterized with a stopped-flow apparatus. This equipment is not sensitive in the near-infrared, allowing the characterization of the exchange kinetics from the increase in the fluorescence of NBD-DPPE only. Figure S14 shows that the rates of exchange increase strongly with temperature for both α_4_ and αβαβ. For consistency in the comparison of the two atropisomers, the characteristic parameters for the rates of exchange shown in Table 1 were obtained from the data for the increase in NBD-DPPE only. The faster exchange rate of αβαβ is mostly due to a higher pre-exponential factor, consistent with its more superficial location in the membrane.

It is important to mention that the time dependencies of the fluorescence intensities in Figure 7 are not strictly monoexponential. A nonexponential variation could indicate a slow translocation of the redaporfin, allowing us to characterize both the rate constant for translocation between the membrane leaflets and desorption from the LUV into the aqueous medium.^18^ However, deviations from a monoexponential dependence may also reflect limitations in the model, such as a slow exchange of the FRET donor, photobleaching, contributions from exchange mediated by collision between LUVs, or sample heterogeneity. In order to attribute physical meaning to the distinct rate constants, it is necessary to perform controls that discard possible artifacts and verify if the relative weight of the distinct exponential steps follows the behavior predicted by the assumed model.^28^ A detailed explanation of the controls performed and results obtained is provided in the Supporting Information (Sections S7–S10).

The rate of exchange is independent of the acceptor-to-donor LUV ratio (Figures S15 and S16) showing that the exchange occurs through redaporfin in the aqueous medium, and not via collision between the donor and acceptor LUVs. Characterization of the LUV samples by dynamic light scattering shows that both the donor and acceptor LUVs have an average diameter slightly above 100 nm (109–119 nm) and a low polydispersity index (0.126–0.148), Figure S17. An estimate of the rate of redaporfin translocation was also obtained through its reduction by dithionite (Figure S18). These results show that the rate-limiting step in α_4_ exchange is its desorption from the membrane, with translocation between the membrane leaflets occurring faster than desorption. In this case, translocation does not influence the rate of exchange between LUVs and a monoexponential behavior was expected. In the case of αβαβ, the results obtained do not allow distinguishing between a faster translocation or desorption and translocation occurring on similar time scales. Nevertheless, the relative weight of the fast and slow components and their dependence on the acceptor/donor ratio do not follow the behavior expected if translocation and desorption occurred on similar (but not equal) time scales, suggesting this was not the origin of the multiexponential behavior observed. The small deviations from a monoexponential behavior are thus attributed mostly to sample heterogeneity (liposomes, redaporfin, and NBD-DPPE, Section S10). The parameters indicated in Table 1 correspond to the weighted average of the rate constants when the results are described by a biexponential function, which are very similar to the rate constant obtained when the overall behavior is obtained from the best fit of a single-exponential.

In order to address the intriguing contrast between the different rates of exchange and similar partition coefficients of α_4_ and αβαβ, we resorted to enhanced sampling MD simulations, which allow for the evaluation of solute permeation through lipid membranes.^12^

### Free Energy of Redaporfin/Membrane Systems

Transition-tempered metadynamics (TT-metaD) simulations allowed the observation of solute permeation and the obtainment of free energy surfaces (FES) for varying CV1 and CV2, as well as the one-dimensional profile across the minimum free energy path (MFEP). Figures S20 and S21 depict the time variation of CV1 during the different TT-metaD simulations. The average number of full translocation events *per* simulation is in the 1–2 range, meaning that while each simulation is probably insufficient in terms of sampling, the consideration of the full set of simulations provides a reasonable number of total translocation events. Additionally, a number of incomplete translocations (where the solute could reach the CV1 = 0 location) were observed, leading to adequate global sampling of the CV space. From these simulations, selected sections and snapshots are presented in Figures S22–S27, illustrating different possibilities of redaporfin permeation events.

**Figure 8:**
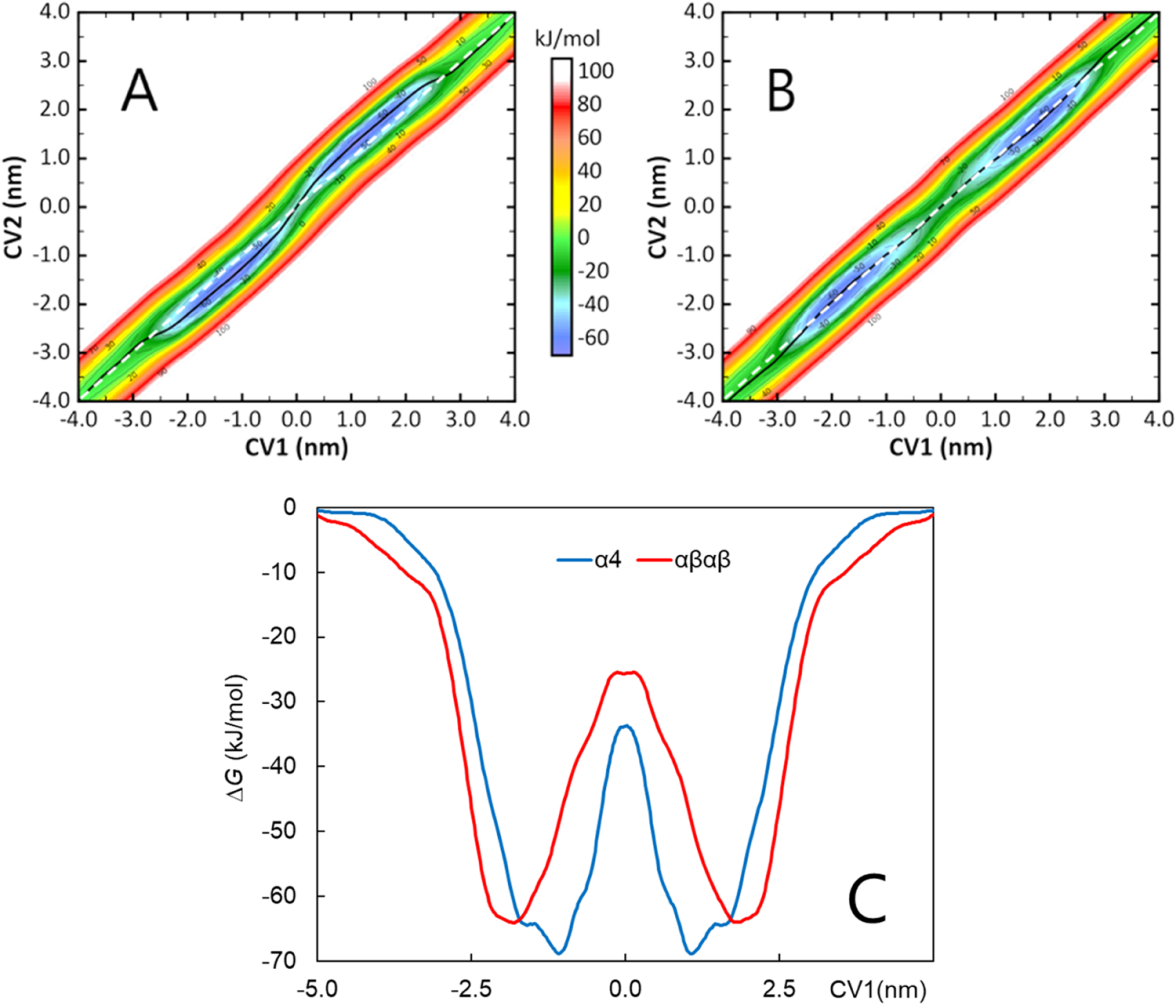
Free energy surfaces calculated from TT-MetaD simulations for α_4_ (A) or αβαβ (B) in the presence of POPC/POPE 4:1 bilayers. The CV1 = CV2 line and the minimum free energy path are shown as dotted white and solid black lines, respectively. (C) One-dimensional free energy profiles of both atropisomers calculated along the minimum free energy path and projected onto the CV1 coordinate. For uncertainty estimates (standard error), see Figure S28.

Figure 8 presents the free energy surfaces (FES) of α_4_ and αβαβ in a POPC/POPE 4:1 bilayer, computed as described in the Materials and Methods section. Physically meaningful FES regions are restricted to a narrow range since CV1 and CV2 are located at no more than 0.51 nm of each other. Despite their correlation, sets of two CVs defined by distances between different solute molecular locations and the center of the bilayer have been previously employed with success to describe solute permeation across lipid bilayers.^29,30^ A different choice for the second collective variable, such as based on local lipid coordinates or local lipid/water densities, could possibly provide fast convergence of the FES.^31,32^ Still, we consider that the choice of CVs taken here is justified by the fact that it provides a readily interpretable mechanistic description of the importance of solute molecular reorientation during the permeation process, which could not be accomplished by replacing CV2 with a lipid- or water-based collective variable. Taking this into account, it is of particular interest to look closer to the region near the CV1 = CV2 identity lines, shown in white dashed lines in Figure 8A,B.

The minimum free energy path (MFEP), shown in black, is virtually indistinguishable from CV1 = CV2 in the αβαβ FES, reflecting the symmetric disposition of the sulfonamide arms attached to the bacteriochlorin ring, which in turn implies that no preferential orientation is observed for αβαβ permeation when the values are averaged over several simulations.

A clear difference is observed for α_4_. For this atropisomer in bulk aqueous medium (|CV1| ≅ 4.0 nm), CV1 and CV2 are almost identical. On approaching the lipid/water interface, despite the mostly random orientation characteristic of the solute in water, a close inspection reveals that |CV2| < |CV1| when 2.8 nm < |CV1| < 3.5 nm. This means that in this distance range, corresponding to the molecule approaching the bilayer, α_4_ tends to orient its sulfonamide substituents toward the lipid headgroups probably because the atropisomer has a large rotational freedom when unattached to the membrane and is driven by the formation of H bonds with the phosphate groups of the lipids. Notably, an inversion in the orientation of α_4_ is observed near |CV1| = 2.8 nm, as |CV2| becomes larger than |CV1| for locations closer to the bilayer center. This means that, after approaching the bilayer with the sulfonamide groups “binding” to the bilayer, the molecule “flips” and, for more internal positions, CV1 has a deeper location than CV2, in agreement with the results from the unrestrained simulations. A second inversion can be clearly seen around the center of the bilayer (|CV1| ≅ 0). In this region, the molecule undergoes a second flipping motion so as to orient the sulfonamide groups toward the lipid/water interface when crossing to the opposite leaflet. At the center, |CV1| and |CV2| are on average identical, indicating a transient molecular orientation with the macrocycle ring perpendicular to the bilayer plane. These MD simulations give full support to the bind-flip mechanism proposed to explain the exception call uptake of α_4_ atropisomer.^9^

The free energy profiles of the two atropisomers, shown in Figure 8C, are relatively similar, although subtle differences are apparent, related to the results discussed above and to the partial curves obtained from the unrestrained simulations (Figure S5). The location of the free energy minimum is more internal, and the internal barrier is narrower, for α_4_ than for αβαβ, in agreement with the average CV1 location determined from the unrestrained MD simulations presented in Figure 5A. Although the difference between atropisomers lies within the estimated uncertainty (see Figure S28), the minimum free energy is 5.5 kJ/mol more negative for α_4_ than for αβαβ, corresponding to , in very good agreement with the experimental value of 6. For both redaporfin atropisomers, the profile along the MFEP shows that the energy barrier for translocation is clearly lower than that for desorption from the membrane. This indicates that desorption is the rate-limiting step in redaporfin exchange between lipid vesicles, which is in agreement with the behavior observed experimentally. It is also observed that, estimated uncertainty notwithstanding, the energy barrier for desorption obtained from the free energy profile (Δ*G*_d_) appears to be larger for α_4_ (Δ*G*_d_ = 64 ± 9 and 69 ± 16 kJ/mol for αβαβ and α_4_, respectively), in agreement with the slower exchange rate constant and higher activation energy (*E*_a_) obtained for this atropisomer (Table 1). However, Δ*G*_d_ is much smaller than that of *E*_a_. This apparent discrepancy between simulations and experiments reflects the fact that they report distinct energy parameters. While the profiles of Figure 8C,D reflect differences in Gibbs free energy, the activation energy obtained experimentally is related to enthalpy variations. The lack of agreement between the two parameters thus reflects a significant variation in the entropy of the system between redaporfin at the equilibrium position in the membrane and in the transition state along the desorption path, Δ^‡^*S*_d_. The very large values obtained for the Arrhenius pre-exponentials indicate a large and favorable entropy variation, in qualitative agreement with the difference observed between Δ*G*_d_ and *E*_a_. The contributions for Δ^‡^S_d_ may be manifold, including changes in the entropy of the membrane, of redaporfin, or reflecting distinct desorption mechanisms and rates of relaxation from the transition state (transmission coefficients).^28,33^ The much lower pre-exponential observed for the desorption of the α_4_ atropisomer may thus reflect the local ordering of the lipid acyl chains near the membrane surface (Figure S11), and the reorientation required for membrane desorption, both leading to a decrease in the transmission coefficient.

Figures S22–S27 illustrate selected permeation events, where reorientation of the solutes is observed both in insertion into and desorption from the lipid bilayer and (for some cases) in their translocation across the two bilayer leaflets.

## Summary and Conclusions

According to the Meyer–Overton rule, cell membrane permeability to a solute is proportional to the membrane affinity of the latter, typically estimated via its partition coefficient from water to organic solvents like *n*-octanol.^10^ This paradigm, foundational to Lipinski’s “rule of five” for drug absorption,^34^ emphasizes overall solute properties. However, our study challenges this approach using two redaporfin atropisomers, with identical chemical bonds but differing in PDT efficiency.^9^

Our results reveal four significant limitations in the use of current predictive approaches to membrane permeability. First, atropisomer membrane partition coefficients exceed their *n*-octanol/water partition coefficients by several orders of magnitude. This discrepancy, noted especially for charged solutes,^35,36^ extends here to uncharged molecules. Second, a significantly higher partition coefficient to lipid membranes was observed for atropisomer α_4_, despite both atropisomers displaying similar *n*-octanol–water partition coefficients .^9^ This differential change highlights the need to consider 3D solute properties in its interactions with lipid membranes. Previous studies have demonstrated the role of conformation-dependent properties, which modulate the exposure of polar and nonpolar groups (chameleonic behavior),^37,38^ and facilitate intramolecular hydrogen bonding.^39−41^ However, redaporfin atropisomers are predominantly rigid, precluding intramolecular hydrogen bonding and underscoring the significance of other 3D properties, particularly their amphiphilicity.^23,42−44^ Third, the rate of exchange between membranes is slower for the atropisomer with the highest membrane affinity. The expectation from Meyer–Overton rule is that the rate-limiting step of membrane exchange is translocation through the nonpolar core of the membrane. Although previous work^43,45−48^ has highlighted that this is a limitation when comparing the behavior of compounds of distinct lipophilicity, we now demonstrated rate-determining membrane desorption kinetics for solutes with identical chemical bonds, ruling out variations in molecular structure as a confounding factor. The slow exchange rate of α_4_ underscores the need to assess solute desorption kinetics to understand equilibration in complex membrane-rich environments as observed *in vivo*. Fourth, stronger interactions with the membrane, measured by relative partition coefficients (*K*_P_), were shown to be associated with much slower exchange rates (*k*) of redaporfin atropisomers. Exchange rates also reflect the strength of the interactions with the membrane, but they are much more sensitive than partition coefficients: the *K*_P_ of α_4_ is a factor 6.5 higher than that of αβαβ but its *k* is 127 times lower. In summary, accurate prediction of drug equilibration rates between lipid membranes requires accounting for their specific interactions in aqueous and lipid environments as well as at their interface.

Interestingly, the MD simulations conducted to obtain insight into the mechanism of α_4_ permeation support the ’bind-flip’ mechanism proposed for cellular internalization of amphiphilic macromolecules. This mechanism facilitates the interaction of the α_4_ atropisomer with the membrane, increases its binding affinity, and leads to a deeper location in the membrane which facilitates its translocation through the membrane core. In contrast, a lower affinity and more superficial location is observed for the symmetric αβαβ atropisomer, leading to a membrane translocation by simple diffusion. Solute asymmetry can thus have a large effect on transport dynamics.

In conclusion, accurate prediction of drug–membrane transport requires a nuanced approach incorporating solute-membrane and solute-water interactions as well as dynamic 3D properties. This work provides a framework for advancing membrane permeability predictions, potentially refining drug design strategies.

## Materials and Methods

### Materials

The lipids 1-palmitoyl-2-oleoyl-*sn*-glycero-3-phosphocholine (POPC), 1-palmitoyl-2-oleoyl-*sn*-glycero-3-phosphoethanolamine (POPE), and 1,2-palmitoyl-*sn*-glycero-3-phosphoethanolamine labeled with 7-nitro-2,1,3-benzoxadiazol-4-yl in the ethanolamine group (NBD-DPPE) were acquired from Avanti Polar Lipids, Inc. (Alabaster, AL). Redaporfin was prepared and purified as previously described.^9^ The water used to prepare the solutions was first distilled and then deionized by ion exchange cartridges and activated carbon filters (ARIOSO UP, from Human, Seoul, Republic of Korea), with a final resistance of ≥ 18 MΩ. Other reagents and solvents used were analytical-grade or of higher purity.

### Preparation of Large Unilamellar Vesicles (LUVs)

The LUVs were prepared by extrusion as previously described.^23^ Briefly, a lipid film was slowly hydrated with the aqueous buffer (phosphate 10 mM at pH 7.4 containing 150 mM NaCl), the multilamellar vesicles formed were subject to several cycles of freeze–thaw and then extruded through two stacked polycarbonate filters with 100 nm pore size. The liposomes were allowed to stabilize overnight before use, to allow for the dissipation of the stress imposed by extrusion,^49^ and their effective size was characterized by dynamic light scattering (Zetasizer Nano ZS, Malvern). The average diameter was 105 nm for POPC LUVs, 110 nm for POPC:POPE 4:1 blank LUVs, containing 2 mol % NBD-DMPE, and slightly larger for LUVs containing also 0.5 mol % redaporfin (132 and 135 nm for α_4_ and αβαβ, Figure S17). The final lipid concentration was quantified by using the Bartlett method. The liposome samples were stored at 4 to 8 °C and used within 2 weeks.

### Partition of Redaporfin to the LUVs

Stock solutions of the redaporfin isotopomers α4 and αβαβ were prepared in DMSO and quantified by UV–vis absorption assuming a molar absorptivity of 1.2 × 10^5^ M^–1^ cm^–1^ at 748 nm.^9^ The required volume of the stock solutions was added to LUV suspension at different lipid concentrations previously added to 96-well plates or quartz cuvettes and preheated at 37 °C. The plate/cuvettes were covered and mixed to achieve solution homogeneity. The concentration of the stock redaporfin solutions was adjusted to obtain the selected final concentration (2.1–50 nM) and 5% DMSO. The fluorescence spectra and/or the fluorescence intensity at λ_exc_/λ_em_ = 508/752 nm was recorded over time in a Cary Eclipse spectrofluorimeter (Varian, Singapore) or a SpectraMax iD5 plate reader (Molecular Devices, Berkshire, U.K.).

The partition coefficient (*K*_P_) was calculated from the dependence of the fluorescence intensity at 752 nm with the lipid concentration, assuming that all the lipid is available to redaporfin, eq 1:
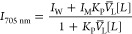
1where *I*_W_ and *I*_M_ are the fluorescence intensity obtained when all redaporfin is in the aqueous medium or associated with the membrane, respectively. The molar volume (*V̅*_L_) considered was 0.8 dm^3^/mol for both POPC and POPE.^50^ The statistical difference between the partition coefficients obtained for the two atropisomers for the same lipid composition or for the same atropisomer between different lipid compositions was evaluated through the *p*-value, calculated using the T.TEST function of Microsoft Excel considering all estimates of the experimental variables, two-tailed distribution, and two-sample unequal variance.

### Rate of Redaporfin Exchange between POPC:POPE LUVs

LUVs containing POPC:POPE at the molar ratios 4:1, 1 mol % NBD-DMPE and 0.5 mol % redaporfin (donor LUVs) were prepared following the procedure indicated above after mixing all components from their stock solutions in chloroform (POPC and POPE), methanol (NBD-DMPE) or ethanol (redaporfin), and allowing for equilibration with occasional vortex during 1 h. Donor LUVs were mixed with PBS or with blank LUVs to obtain a final concentration of lipid in donor LUVs equal to 0.1 mM and acceptor:donor ratios of 0, 1, 3, and 6.5 (all solutions pre-equilibrated at the required temperature), and the time evolution of redaporfin and NBD-DPPE fluorescence (λ_exc_/λ_em_ = 460/752 or 460/530 nm, respectively) was followed in a Cary Eclipse spectrofluorimeter. Due to the fast exchange of αβαβ, the fluorescence variation was also followed in SF-61 stopped-flow equipment (Hi-Tech, Salisbury, U.K.), with excitation at 450 nm and fluorescence emission collected with a 520 nm cutoff filter. The stopped-flow detector is not sensitive at the wavelengths of redaporfin fluorescence emission, the fluorescence variation thus reporting only the increase in NBD fluorescence due to exchange of redaporfin to the acceptor LUVs. The rate constant of redaporfin exchange was obtained from the best fit of a mono- or biexponential function to the time dependence of the fluorescence intensity. When the introduction of the second exponential decreased the χ^2^ of the best fit to half or less, the exchange rate constant was obtained from the weighted average, eqs 2 and 3. Otherwise, the exchange rate constant was obtained from the best fit of a monoexponential function.
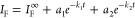
2
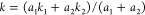
3Complementary experiments were performed with the transfer of redaporfin from donor LUVs containing only redaporfin at 0.5 mol % to acceptor and acceptor LUVs containing 1 mol % NBD-DPPE symmetrically distributed on both membrane leaflets, or to asymmetric LUVs containing NBD-DPPE in the inner leaflet only obtained by reaction of with dithionite.^27^ Statistically equivalent results were obtained in all situations, with all of the results being included in the average rate constants shown in Table 1.

### Unrestrained MD Simulations

MD simulations and analysis of trajectories were carried out using GROMACS 2019,^51^ with the Amber force field. For starting structures, mixed POPC/POPE (4:1) bilayers were built and hydrated using the Membrane Builder tool from CHARMM-GUI.^52^ Phospholipid parameters were taken from the Slipids force field.^53,54^ The original TIP3P model was used for water, where the Lennard-Jones parameters for the hydrogen atoms are equal to zero.^55,56^ Redaporfin atropisomers α_4_ and αβαβ were parametrized using a procedure previously used by one of us.^57^ The parametrization was made using Generalized Atomic Force Field (GAFF),^58^ which is compatible with the Amber force field. First we optimized the geometry and then used the RESP procedure implemented in R.E.D.^59^ to obtain the partial charges on all atoms. The next step was to obtain the missing terms in the force field, for which ACPYPE was used.^60^ All topology files used are available at https://github.com/peabreu/Redaporfins. For unrestrained simulations, 130 phospholipid bilayers (104 POPC:26 POPE), with 5705 water molecules and two molecules of either α_4_ or αβαβ, initially located near the water-membrane interface of each bilayer leaflet, were prepared and simulated in triplicate. The membrane systems were equilibrated with the CHARMM-GUI protocol, which included a minimization step and several small NVT and NPT equilibration steps with position restraints, which were gradually alleviated until the restraint-free 1 μs production runs.^61^ For analysis, unless stated otherwise, the last 750 ns of each simulation were considered.

These simulations were performed under NPT conditions, with an integration step of 2 fs. Constraints in the H bonds were applied using the LINCS algorithm.^62^ Dispersion corrections were used for both the potential and pressure. Periodic boundary conditions were applied. The electrostatics interactions were modeled with the particle-mesh Ewald (PME) method.^63^ Both real-space Coulomb and van der Waals potentials were switched to zero values at a 1.4 nm cutoff. For temperature control, membrane lipids (together with redaporfin atropisomers) and water molecules were coupled independently, with temperature baths at 310.15 K using the Nose–Hoover algorithm^64,65^ with coupling time constant of 0.5 ps. The pressure was maintained constant at 1.013 bar with a barostat employing the Parrinello–Rahman algorithm^66^ with a semi-isotropic scheme, a coupling constant of 2.0 ps, and a compressibility of 4.5 × 10^–5^ bar^–1^.

### Enhanced Sampling MD Simulations

Enhanced sampling simulations employing the TT-MetaD method^67^ were conducted using GROMACS 2019, patched with the PLUMED library (version 2.5.7).^68,69^ This method has been demonstrated to be effective in determining the free energy surface of solutes permeating a lipid bilayer.^29^

TT-MetaD simulations were carried out with a 104 POPC:26 POPE bilayer, hydrated with 6400 water molecules, previously equilibrated in a 500 ns unrestrained simulation, run as described above. One redaporfin molecule (either α_4_ or αβαβ) was then placed in the water medium, and the system was simulated by applying energy bias along two collective variables (CVs). The first CV was defined as the transverse distances between the center of mass (COM) of the bilayer and that of the four macrocycle N atoms (CV1). The second CV was defined as the transverse distance of the two diametrically opposite S atoms (CV2); see Figure 3 for illustration. These distances, which define the CV space, were normalized during the simulation in order to obtain the proper periodicity in the CV space to account for the fluctuations in the simulation box under the NPT conditions. The normalization range was set to [−0.5, 0.5]. The bias energy added to the CV space follows a Gaussian curve. The initial height of the Gaussian hill was set as 0.015 kJ/mol and the width (σ) as 0.02 in normalized coordinates for both dimensions, which corresponds approximately to 0.22 nm in our system. The pace of bias deposition was set to every 500 steps. The TT-metaD basins were defined at (0.0, 0.0) and (0.48, 0.48) in the CV space. When a transition path occurred in the system, the Gaussian height was tempered using a bias factor (γ) of 30 after exceeding a bias threshold of 10 kJ/mol. All other MD run options were as described above for the unrestrained simulations.

For each system, multiple (4 for α_4_, 6 for αβαβ) 700 ns independent replicates were simulated and analyzed. The resulting free energy surfaces in (CV1, CV2) space were then averaged and symmetrized. In order to redefine the CV dimensions as absolute distances, the CV values were multiplied by the averaged *z*-box dimensions obtained during these simulations. These surfaces were then used to search for the minimum free energy path (MFEP) along the surface using the string method at zero Kelvin.^70^ To obtain a 1D free energy profile, MFEP was projected onto one of the CV dimensions (arbitrarily chosen as CV1).

## Supporting Information Available

Additional experimental and computational results: electrostatic surface potential and dipole moments of the redaporfins; effect of incubation time and concentration on the apparent affinity for POPC:POPE LUVs; complementary unrestrained MD simulation results; absorption and fluorescence spectra of NBD-DPPE and redaporfin; time dependence of NBD-DPPE fluorescence intensity; effect of temperature and the ratio of acceptor/donor LUVs on the rate of redaporfin exchange, liposome size, and polydispersity; rate of redaporfin reduction by dithionite; evaluation of NBD-DPPE purity; and selected permeation events from the TT-MetaD simulations (PDF)
