# Supplementary material for: Passive Transport across Cell Membranes beyond the Overton Rule: Insights from Solute Exchange in Vesicles and Molecular Dynamics of Atropisomers
Source: ACS Appl Mater Interfaces. 2025 Apr 10;17(16):23575–87. doi: 10.1021/acsami.4c22459 (PMC12022943; doi:10.1021/acsami.4c22459)
Supplement: Supplementary file 1 — am4c22459_si_001.pdf [file am4c22459_si_001.pdf]

Supporting Information for

Passive Transport Across Cell Membranes Beyond the Overton Rule: Insights from Solute Exchange in Vesicles and Molecular Dynamics of Atropisomers

Margarida M. Cordeiro,[1,2,#] Alexandre C. Oliveira,[1,2,#] Paulo E. Abreu,[1,2] Luis G. Arnaut,[1,2] Maria João Moreno,[1,2,3,*] Luís M. S. Loura[1,3,4,*]

[1] Coimbra Chemistry Center, Institute of Molecular Sciences (CQC-IMS), University of Coimbra, 3004-535 Coimbra, Portugal
[2] Department of Chemistry, University of Coimbra, 3004-535 Coimbra, Portugal
[3] CNC—Center for Neuroscience and Cell Biology, University of Coimbra, 3004-535 Coimbra, Portugal
[4] Faculty of Farmacy, University of Coimbra, 3000-548 Coimbra, Portugal

[*] Corresponding authors. E-mail addresses: mmoreno@ci.uc.pt (M.J.M.), lloura@ff.uc.pt (L.M.S.L.)
[#] M.M.C. and A.C.O. have contributed equally to this work.

Contents

Section S1 – Electrostatic surface potential and dipole moments of the redaporfins (Figure S1) S2

Section S2 – Effect of incubation time and concentration on the apparent affinity for POPC:POPE LUVs S3
Figure S2 – Effect of incubation time S3
Figure S3 – Effect of redaporfin concentration S4

Section S3 – Complementary unrestrained MD simulation results S5
Figure S4 – Time evolution of the transverse location of redaporfin S5
Figure S5 – Relative density and partial free energy profiles S6
Figure S6 – Angular distribution of redaporfin tilt angle S6
Figure S7 – Time variation of the instant redaporfin dipole moment S7
Figure S8 – Correlation between redaporfin dipole moment and location S7
Figure S9 – Orientation of redaporfin dipole moment S8
Figure S10 – Angular distribution of the bacteriochlorin ring and dipole moment S8
Figure S11 – Acyl chain order parameter profiles at different distances from redaporfin S9

Section S4 – Absorption and fluorescence spectra of NBD-DPPE and redaporfin (Figure S12) S10

Section S5 – Time dependence of NBD-DPPE fluorescence intensity (Figure S13) S11

Section S6 – Effect of temperature on the rate of redaporfin exchange (Figure S14) S12

Section S7 – Effect of the ratio of acceptor/donor LUVs on the rate of redaporfin exchange S13
Figure S15 – Amplitude of fluorescence variation and exchange rate constant of α_4 S13
Figure S16 – Amplitude of fluorescence variation and exchange rate constant of αβαβ S14

Section S8 – Liposome size and polydispersity (Figure S17) S15

Section S9 – Rate of redaporfin reduction by dithionite (Figure S18) S17

Section S10 – Evaluation of NBD-DPPE and redaporfin purity S18
Figure S19 – TLC-RP18 analysis of NBD-DPPE samples S18
Section S11 – Selected permeation events from the TTMetaD simulations S20
Figure S20 and S21 – Variation of the CV1 coordinate for all TTMetaD simulations S20
Figure S22 and S23 – Snapshots and α_4 coordinates during one of the full permeation events S22
Figure S24 to S27 – Snapshots and αβαβ coordinates during two of the full permeation events S23
Figure S28 – One-dimensional free energy profiles along the MFEP, with uncertainty estimates S23

References S27

Section S1 – Electrostatic surface potential of the redaporfins.

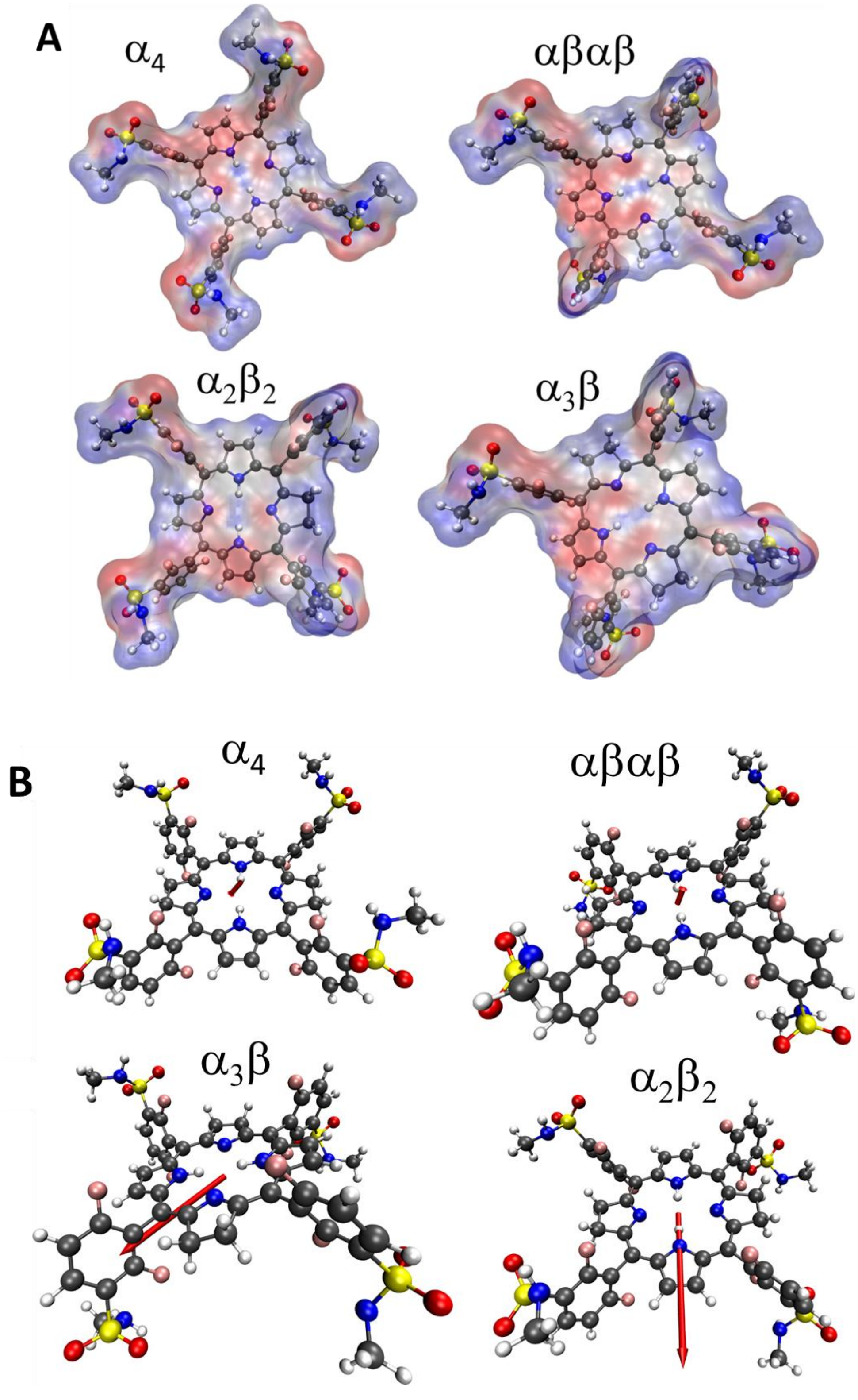

Figure S1. **Plot A**: Molecular electrostatic potential for the four atropisomers at the B3LYP/6-31G(d,p) level of theory. The red corresponds to the negative part and blue to the positive values of the potential. **Plot B**: Illustration of the orientation and magnitude of the dipole moments for the four atropisomers calculated at the B3LYP/6-31G(d,p) level of theory. The arrow points towards the negative pole of the dipole moment.

Section S2 – Effect of incubation time and concentration on the apparent affinity of redaporfin for POPC:POPE 4:1 LUVs.

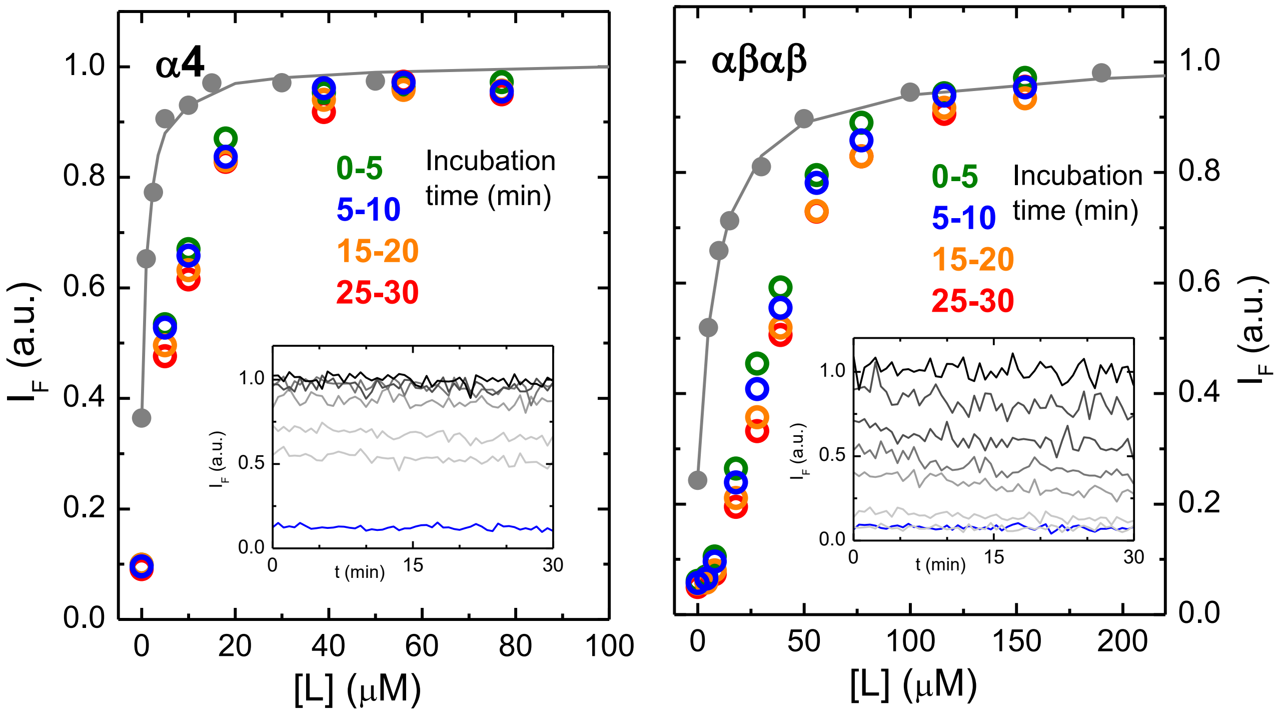

Figure S2 – Effect of incubation time on the variation of redaporfin fluorescence intensity in the presence of LUVs prepared with POPC:POPE 4:1, at 37 °C with 5% DMSO for a redaporfin concentration of 25 nM, for α_4 (left plot) and αβαβ (right plot). The grey filled circles are the experimental results at 2.1 nM redaporfin, and the grey line is the corresponding best fit of a simple partition. The insets show the variation of the fluorescence intensity with the incubation time for a redaporfin concentration of 25 nM, in the absence of lipid (—), at 150 μM lipid (—), and at intermediate lipid concentrations (different tones of gray).

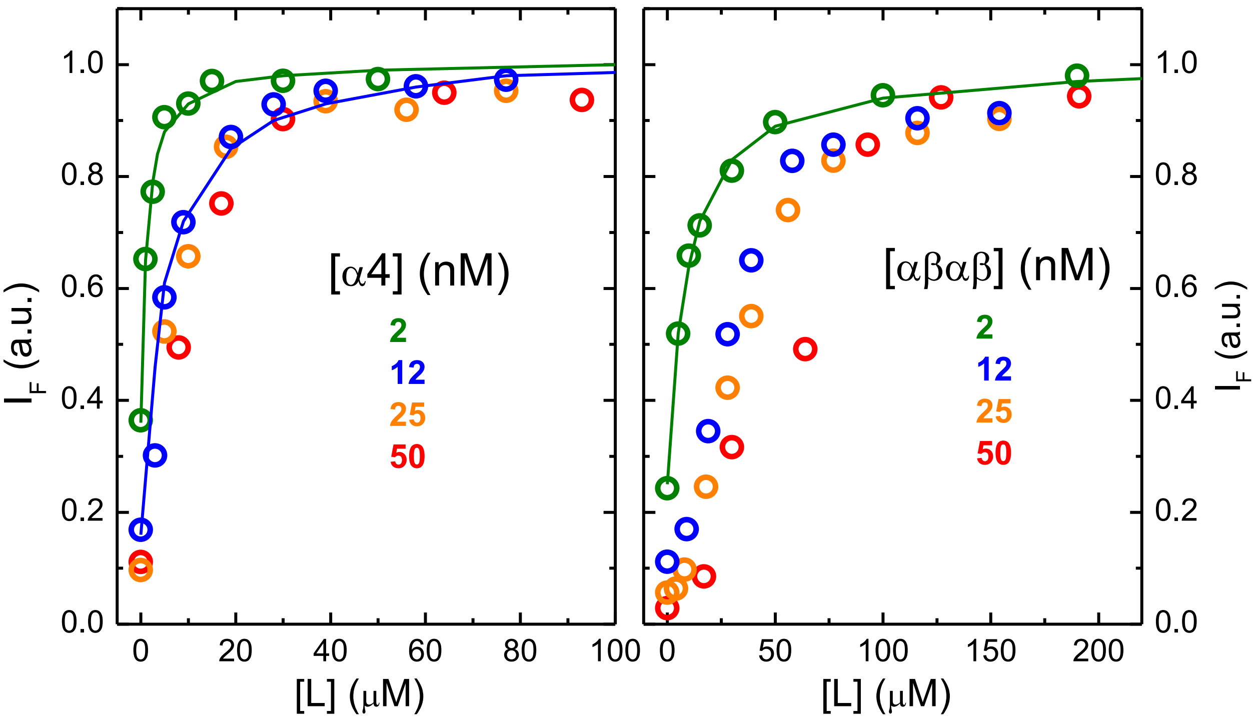

Figure S3 – Effect of redaporfin concentration on the variation of the fluorescence intensity in the presence of LUVs prepared with POPC:POPE 4:1, at 37 °C with 5% DMSO after incubation for 5 min, for α_4 (left plot) and αβαβ (right plot). The lines correspond to the best fit assuming simple partition, equation 1 in the main manuscript.

Section S3 – Complementary unrestrained MD simulation results.

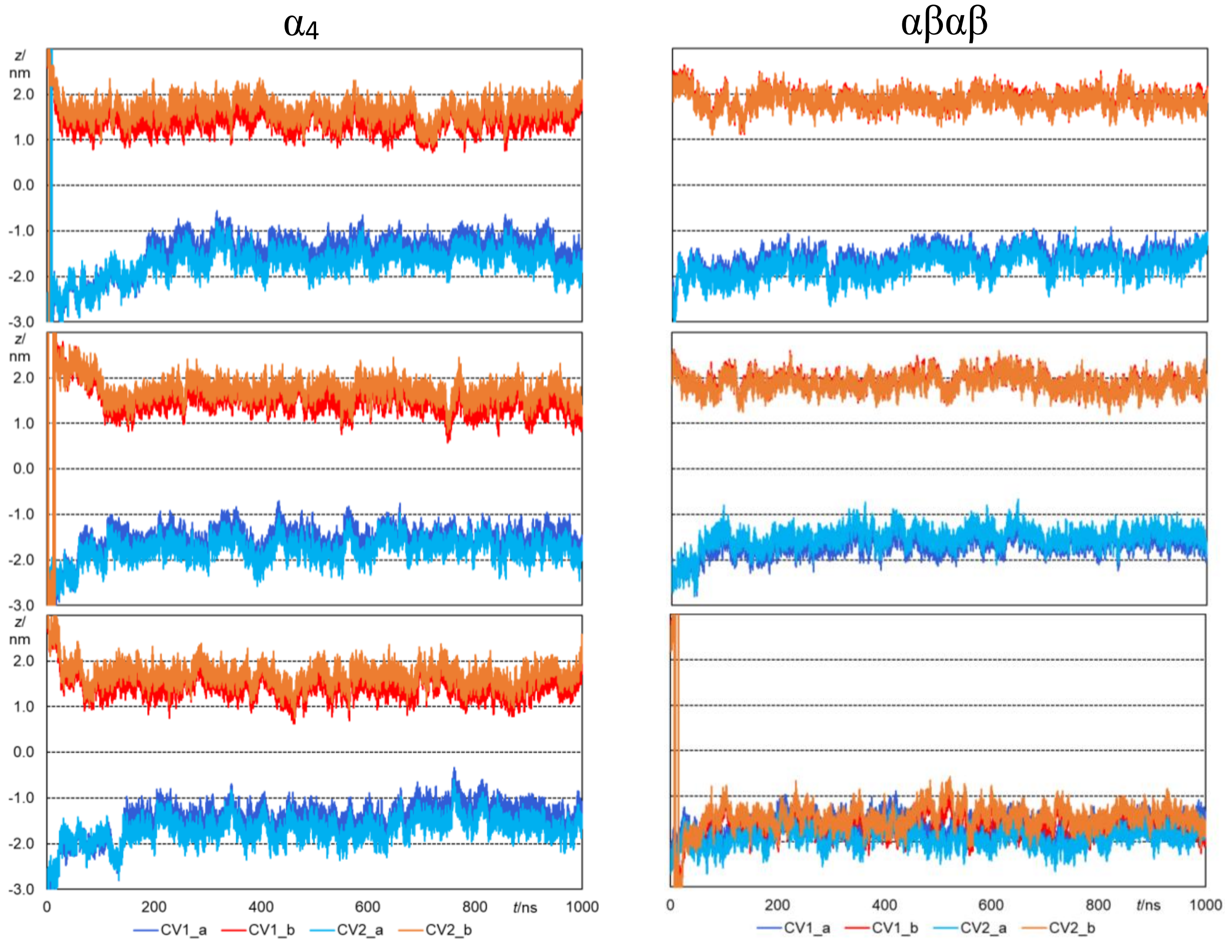

Figure S4. Time evolution of the transverse positions *z* of the collective variables CV1 and CV2 of each individual molecule in the unrestrained MD simulations of redaporfin atropisomers α_4 (left) and αβαβ (right). Each panel concerns a different simulation of two redaporfin molecules interacting with POPC/POPE 4:1 bilayers.

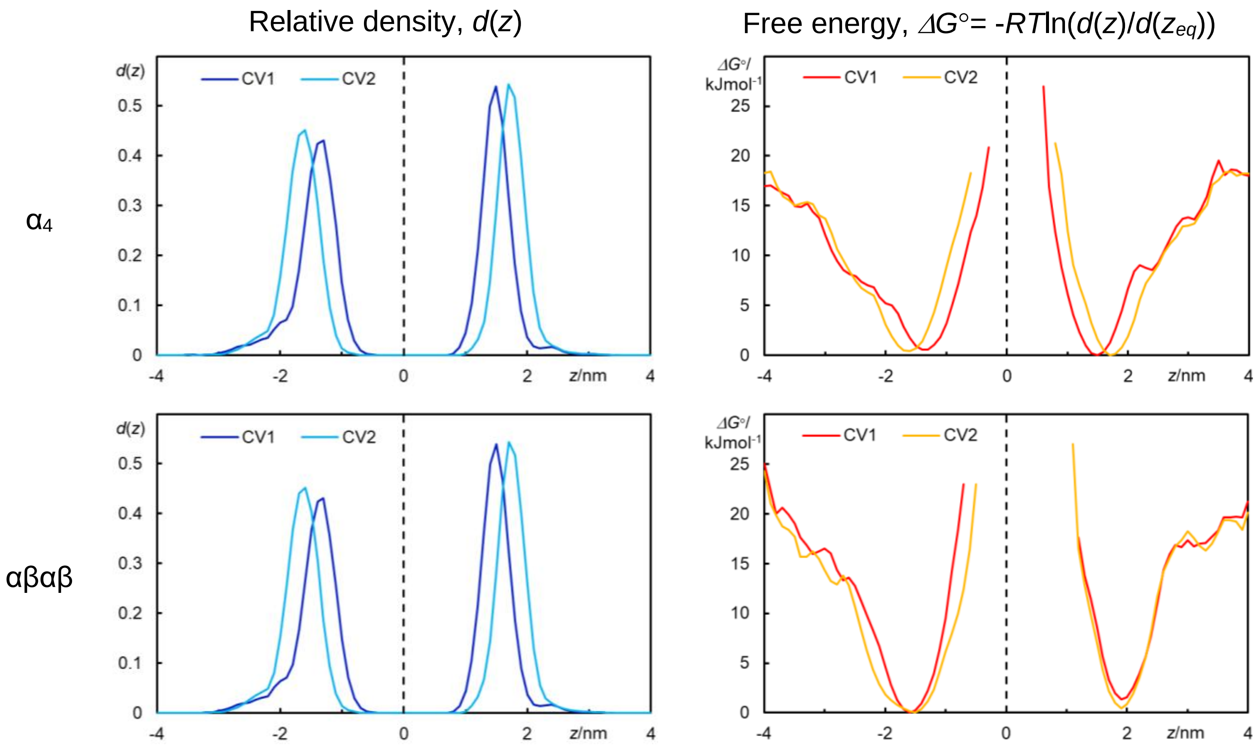

Figure S5 – Relative density and partial free energy profiles associated with the collective variables CV1 and CV2 of the two redaporfin atropisomers, obtained from the unrestrained simulations.

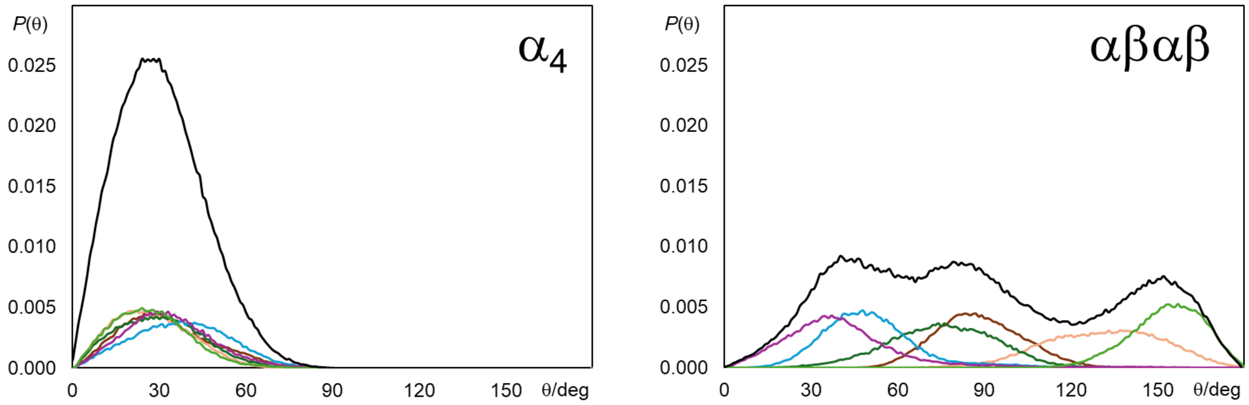

Figure S6 – Angular distribution of the tilt of the CV1 → CV2 vector relative to the bilayer normal for the two atropisomers. The black line is the overall distribution considering all six molecules (also shown in Figure 6B), whose individual distributions are depicted in the colored lines.

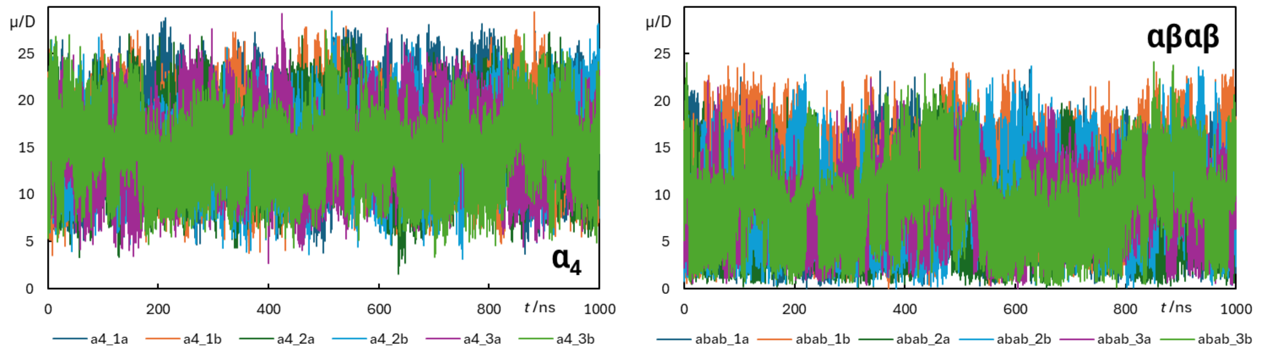

Figure S7 – Time variation of instant redaporfin dipole moment, calculated from the unrestrained simulations. Each line concerns a different molecule (two for each of the three simulations, six in total).

The fluctuations in the magnitude of the dipole moment do not correlate with fluctuations in the depth location of the redaporfin bacteriochlorin ring (Figure S8).

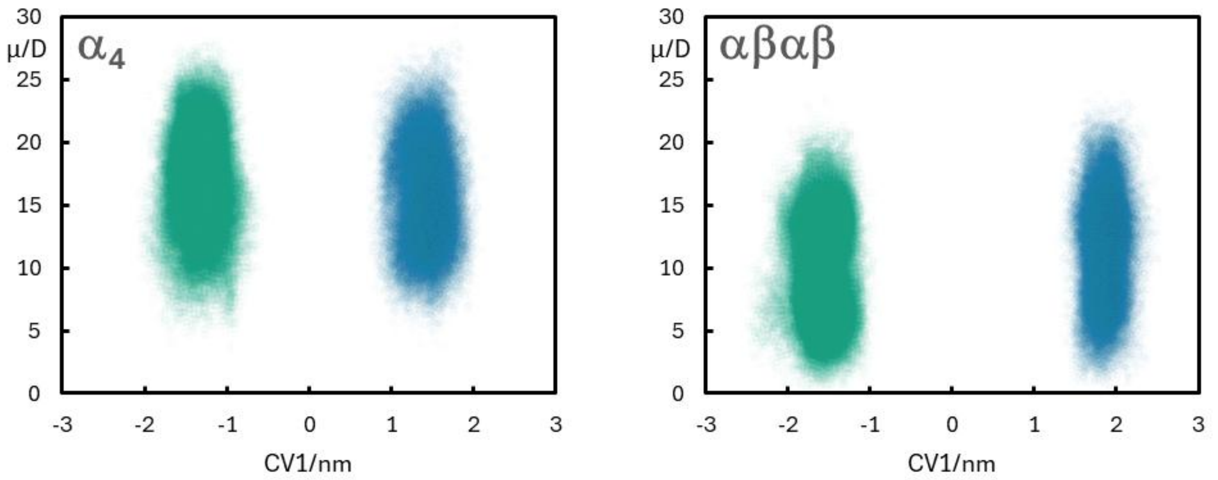

Figure S8 – Correlation between the overall dipole moment of the redaporfin molecule and the depth location of the bacteriochlorin ring in the lipid bilayer. The figure shows the results obtained for the two redaporfin molecules in the first replicate of each atropisomer, equivalent results were obtained for the other two sets of simulations.

The projections of redaporfin dipole moment in the *x*, *y* and *z* axes are shown in Figure S9. The bilayer is aligned with the *x*/*y* plane, and the *z* axis corresponds to the bilayer normal. The data shown are the average of the behaviour of all 6 α_4 molecules, while $\alpha\beta\alpha\beta$ molecules aligned with the bacteriochlorin ring aligned with the bilayer surface (parallel) or inserted in the lipid palisade (perpendicular) were analysed separately. There is a significantly high probability of full alignment of the overall dipole moment of α_4 with the *z* axis, while all projections contribute similarly to the overall dipole in the case of $\alpha\beta\alpha\beta$ aligned parallel to the bilayer surface, and an antiparallel orientation for $\alpha\beta\alpha\beta$ oriented perpendicular to the bilayer surface. Taking into account the orientation of the bacteriochlorin ring when associated with the lipid bilayer, this shows that the dipole moment of α_4 is mostly aligned with the normal to the ring, while that of $\alpha\beta\alpha\beta$ is aligned with the bacteriochlorin ring (Figure S10). The negative pole of α_4 points towards the water, while that of $\alpha\beta\alpha\beta$ is either randomly distributed in the bilayer xy plane, or pointing towards the bilayer center in the case of $\alpha\beta\alpha\beta$ with the normal of the bacteriochlorin ring perpendicular to the bilayer normal. The observation that both $\alpha\beta\alpha\beta$ molecules inserted in the bilayer with the same orientation of the dipole moment suggests the alignment of the dipole moment antiparallel to the leaflet dipole potential contributes significantly to the interactions established between $\alpha\beta\alpha\beta$ and the membrane. This agrees with the observation that the negative pole of the dipole moment is pointing towards the positive dipole potential in the centre of the bilayer. This contributes also to the higher $\log K_{\mathrm{P}}^{\mathrm{PC:PE}}/\log K_{\mathrm{P}}^{\mathrm{PC}}$ observed for $\alpha\beta\alpha\beta$ when compared to α_4, with $\alpha\beta\alpha\beta$ being stabilized and α_4 destabilized by the higher dipole potential of the PC:PE membrane [1-3].

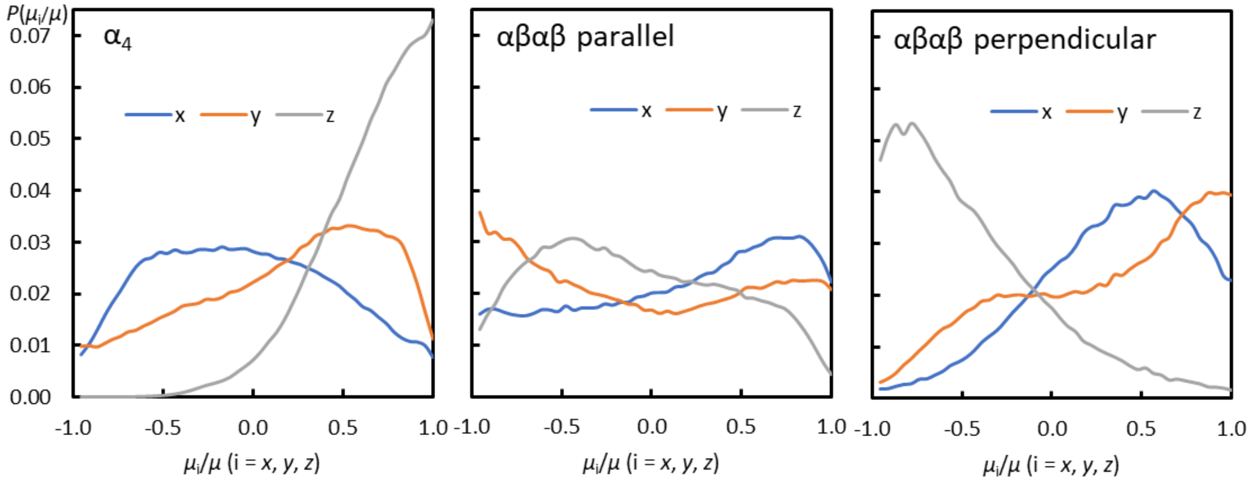

Figure S9 – Probability density functions of the relative projections μ_i/μ of the redaporfin dipole moment along the *x*, *y* and *z* directions, where *xy* is the bilayer plane and *z* the normal direction, respectively.

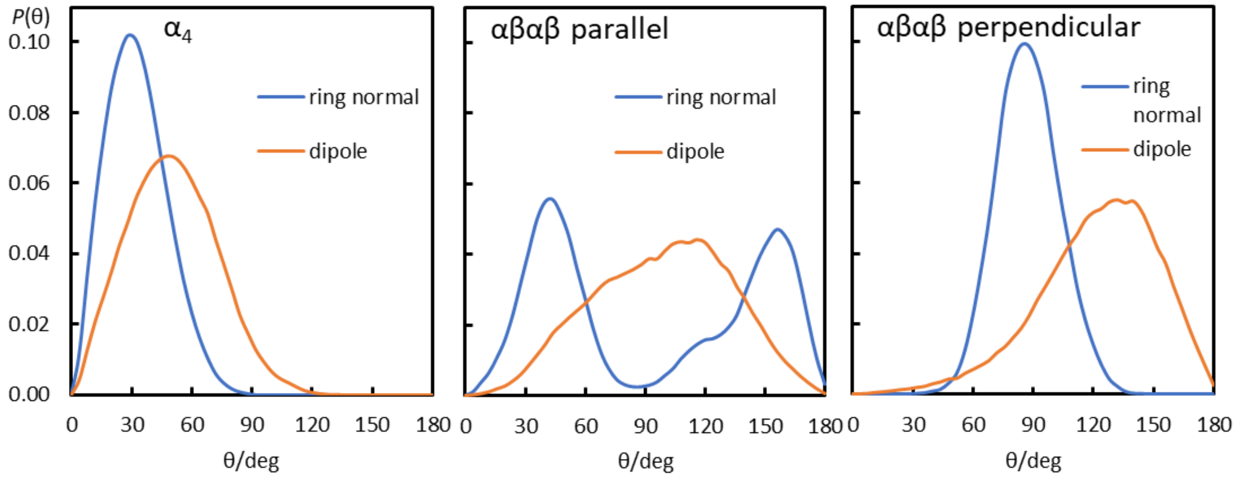

Figure S10 – Probability density functions of the tilts of the redaporfin dipole moment and the normal to the bacteriochlorin ring, relative to the normal to the bilayer plane.

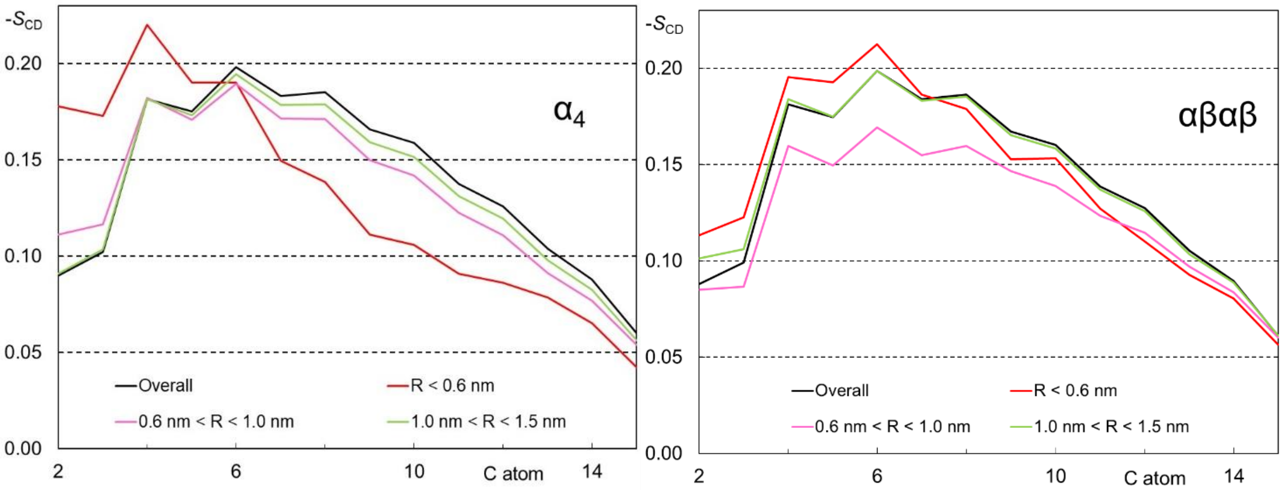

Figure S11 – Acyl chain order parameter profiles -S_{CD}, calculated for different distance *R* between the centers of mass of the phospholipid acyl chain and of the redaprofin molecule inserted in the same leaflet. The profiles corresponding to *R* > 1.5 nm are virtually identical to the overall curve and therefore not shown.

Section S4 – Absorption and fluorescence spectra of NBD-DPPE and redaporfin.

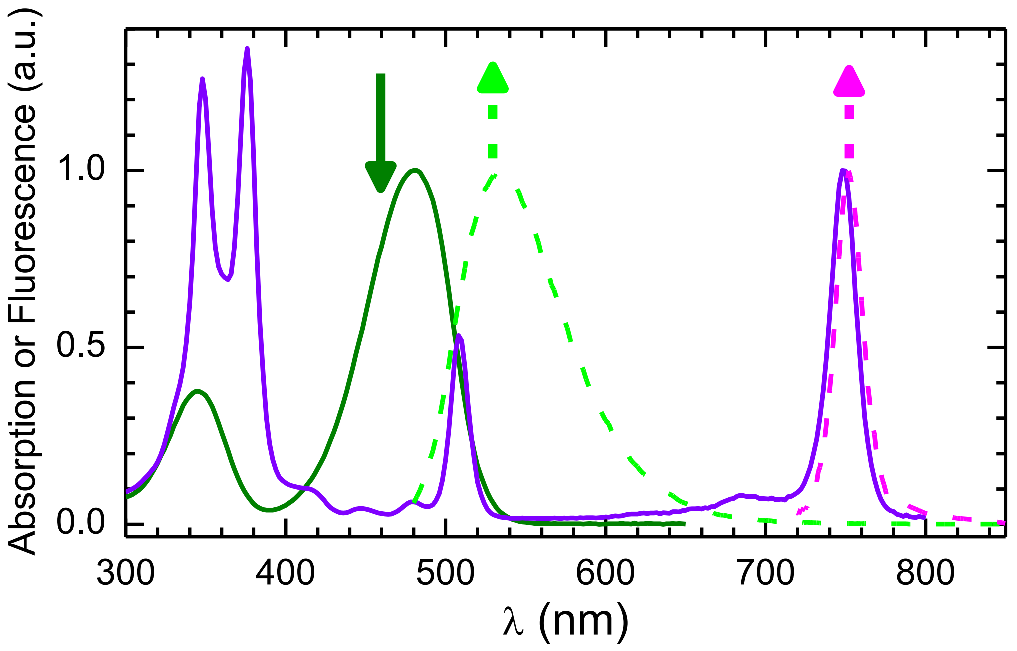

Figure S12 – Absorption and fluorescence spectra of NBD-DPPE and redaporfin atropoisomer α4. The absorption spectra in DMSO are shown as continuous lines (— for NBD-DPPE, and — for α4), and the fluorescence spectra when associated with POPC:POPC 4:1 LUVs is shown by dashed lines (– – for NBD-DPPE, and – – for α4). The wavelength used for excitation in the FRET experiments was 460 nm (indicated by the arrow ↓), while the fluorescence intensity of the FRET donor was collected 530 nm (↑) and that of the FRET acceptor was collected at 752 nm (↑).

An estimate of the FRET Förster radius for the pair NBD-DPPE /redaporfin was calculated from equations below, where R_0 is obtained in Å when the wavelength is in nm and the molar absorptivity of the acceptor (ε_A) in M^{-1} cm^{-1}, equations (S1) and (S2).

$$R_0 = 0.211[k^2 n^{-4} \emptyset_F \, J(\lambda)]^{\frac{1}{6}} \quad (S1)$$

$$J(\lambda) = \frac{\int_0^{\infty} F_D(\lambda)\, \varepsilon_A(\lambda)\, \lambda^4 d\lambda}{\int_0^{\infty} F_D(\lambda)\, d\lambda} \quad (S2)$$

A random orientation of the donor and acceptor molecules was considered (κ^2= 2/3), and the refractive index of methanol was used to describe the medium where the donor and acceptor molecules are located (n = 1.326 [4]). Considering a molar absorptivity of $7.0{\times}10^4$ M^{-1} cm^{-1} [5] for redaporfin at the absorption band near 500 nm, and a fluorescence quantum yield of NBD-DPPE in the membrane equal to 0.32 [6], a value of R_0 = 41 Å is obtained.

Section S5 – Time dependence of NBD-DPPE fluorescence intensity.

The rate of NBD-DPPE exchange was evaluated through the addition of blank LUVs to donor LUVs containing NBD-DPPE at self-quenching conditions (2 mol%). No significant exchange of NBD-DPPE occurred over 60 h at 37 °C. Figure S13 shows the effect of adding blank LUVs to the fluorescence intensity of NBD-DPPE at the same total concentration, but in LUVs containing 2 mol% NBD-DPPE or in LUVs containing 1 mol% NBD-DPPE and 0.5 mol% α_4. At the beginning of the experiment, the fluorescence intensity of NBD-DPPE is smaller in the LUVs containing both NBD-DPPE and α_4 due to efficient FRET. However, in the course of the experiment, the fluorescence intensity of NBD-DPPE in the solution containing both probes becomes higher than that of LUVs containing only NBD-DPPE. This is due to the decrease in NBD-DPPE fluorescence quenching by α_4 due to the exchange of α_4, and to stable self-quenching of NBD-DPPE in the LUVs containing only this probe at a local concentration of 2 mol%. The fluorescence intensity of the LUVs containing only NBD-DPPE is invariant, confirming that the local concentration of NBD-DPPE is maintained, and therefore, that it does not equilibrate with the blank LUVs in the 10 h of the experiment at 37 °C. The experiment was repeated at 55 °C, and again, no significant variation was observed in the fluorescence of NBD-DPPE (lower plot). This figure also shows that there is an increase in the fluorescence intensity when LUVs containing 2 mol% NBD-DPPE are mixed with blank LUVs in the presence of 0.5 mM methyl β-cyclodextrin (MeβCD). At this low concentration MeβCD does not disrupt LUVs (verified by Dynamic Light Scattering), but interacts with the membranes promoting lipid exchange. The exchange of NBD-DPPE to the blank LUVs leads to a lower local concentration, and therefore to a decrease in self-quenching with an increase in fluorescence intensity.

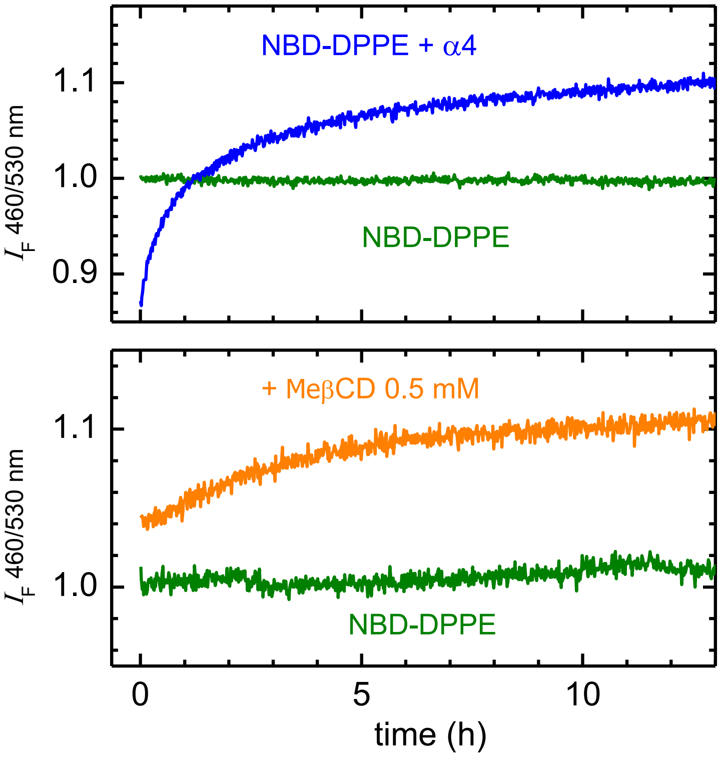

Figure S13 – Time dependence of the fluorescence intensity from NBD-DPPE. Upper plot – LUVs containing 2 mol% NBD-DPPE (—) or containing 1 mol% NBD-DPPE and 0.5 mol% α4 (—) were mixed with excess (1:3) blank LUVs at 37 °C. Lower plot – LUVs containing NBD-DPPE at 2 mol% were mixed with excess (1:3) blank LUVs at 55 °C, in the absence (—), and presence of 0.5 mM MeβCD (—).

Section S6 – Effect of temperature on the rate of redaporfin exchange between POPC:POPE 8:2 LUVs.

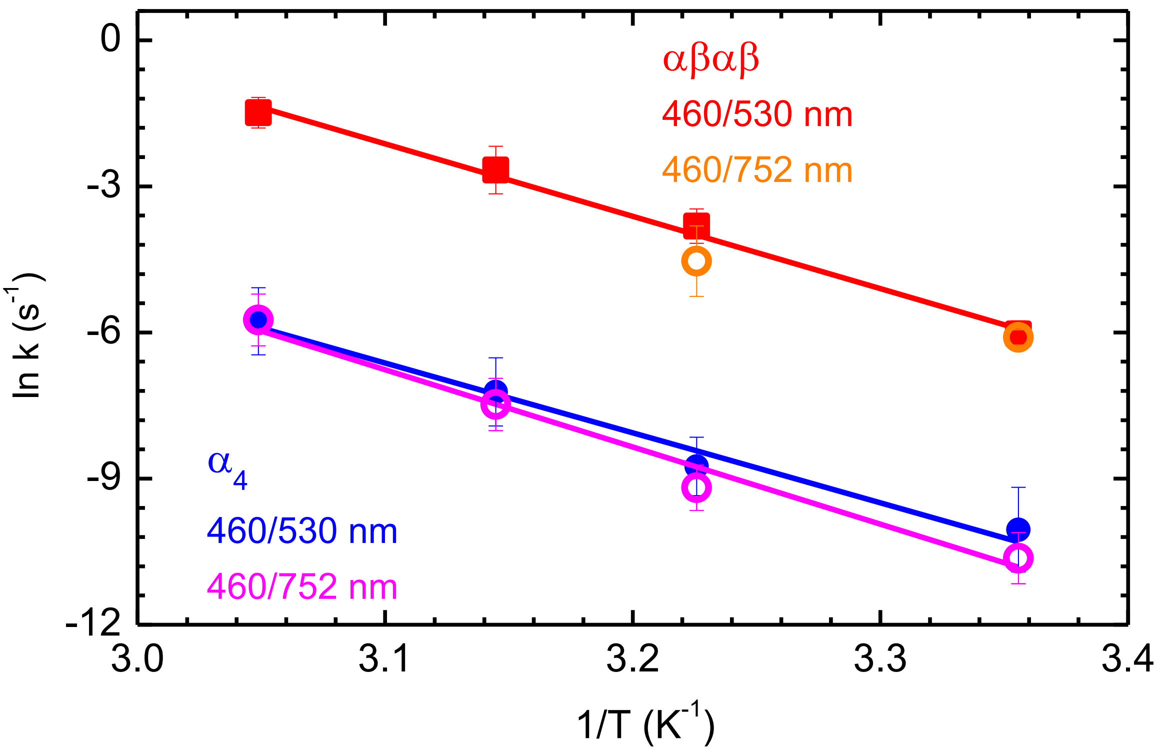

Figure S14 – Temperature dependence of the rate of exchange of α_4 and αβαβ when followed by the increase in the fluorescence of the donor ($\lambda_{ex}/\lambda_{em}$=460/530 nm, filled symbols), or from the decrease in the fluorescence of the acceptor ($\lambda_{ex}/\lambda_{em}$=460/752 nm, hollow symbols). The lines are the best fit of the Arrhenius equation, with the parameters discussed in the text and shown in Table 1 of the main text.

Section S7 – Effect of the ratio of acceptor/donor LUVs on the rate of redaporfin exchange.

The exchange of redaporfin from POPC:POPE 4:1 LUVs containing 1 mol% NBD-DPPE to blank POPC:POPE 4:1 LUVs, was followed at a lipid concentration of donor LUVs fixed at 0.1 mM and variable concentrations of blank acceptor LUVs. The effect of the ratio of acceptor/donor LUVs in the amplitude of fluorescence variation and on the rate of exchange is shown below, average and standard deviation of 3 experiments for the exchange of α_4 atropoisomer.

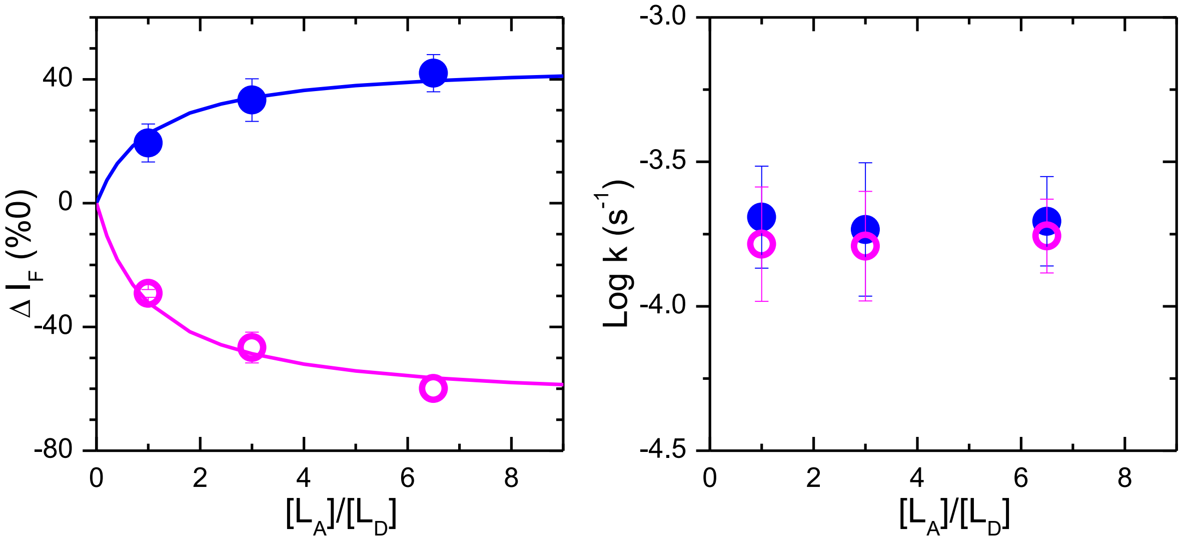

Figure S15 – Dependence of the magnitude of fluorescence variation (left plot) and average exchange rate constant (right plot) on the ratio of acceptor-to-donor LUVs, for the exchange of α_4 between POPC:POPE 4:1 LUVs at 37 °C. The results obtained from the variation of NBD-DPPE fluorescence (λexc=460/530 nm) are shown by filled circles in blue, and those from the variation of α_4 fluorescence (λexc=460/752 nm) are shown as open circles in magenta. The lines in the left plot correspond to the best fit of equations (S3) to (S5).

As expected, as the ratio of acceptor-to-donor LUVs increases, the amplitude of fluorescence variation increases as well. The behavior is well described by equation S5, which assumes full equilibration during the time window analyzed, and the same affinity of α4 between the donor and acceptor LUVs.

$$[\alpha 4]_{\mathrm{D}}(0) = [\alpha 4]_{\mathrm{T}}(0)\frac{\overline{V_{\mathrm{L}}}K_{\mathrm{P}}[L]_{\mathrm{D}}}{1+\overline{V_{\mathrm{L}}}K_{\mathrm{P}}[L]_{\mathrm{D}}} \quad \text{(S3)}$$

$$[\alpha 4]_{\mathrm{D}}(\infty) = [\alpha 4]_{\mathrm{T}}(0)\frac{\overline{V_{\mathrm{L}}}K_{\mathrm{P}}[L]_{\mathrm{D}}}{1+\overline{V_{\mathrm{L}}}K_{\mathrm{P}}([L]_{\mathrm{D}}+[L]_{\mathrm{A}})} \quad \text{(S4)}$$

$$\Delta I_{\mathrm{F}}(\infty,[L]_{\mathrm{A}}) = \Delta I_{\mathrm{F}}(\mathrm{max})\{[\alpha 4]_{\mathrm{D}}(\infty) - [\alpha 4]_{\mathrm{D}}(0)\} \quad \text{(S5)}$$

From the best fit one obtains the maximum variation in the fluorescence intensity, which corresponds to the signal in the absence of energy transfer from NBD-DPPE to redaporfin. In the case of NBD-DPPE, $\Delta I_{\mathrm{F}}(\mathrm{max})$= 47 ± 7 % was obtained, indicating that its fluorescence is being efficiently quenched by FRET to redaporfin, in good agreement with the high R_0 calculated for this FRET pair. The maximal decrease obtained for redaporfin fluorescence intensity when excited at 460 nm was 68 ± 4 %, indicating that redaporfin fluorescence for λexc=460 nm is significant even without FRET from NBD-DPPE. In fact, although the relative absorption of α_4 at 460 nm is lower than that of NDP-DMPE, it is not as negligible as suggested by Figure S4 due to the much higher molar absorptivity of redaporfin. When considering their molar absorptivity and the concentrations used in the exchange experiments, around 10 % of the excitation light absorbed is by redaporfin directly. The fraction of redaporfin directly excited is somewhat higher because NBD-DPPEquantum yield and the FRET efficiency are significantly lower than one.

In the case of αβαβ, the effect of the acceptor-to-donor ratio on the amplitude of fluorescence variation of both the donor (NBD-DPPE) and acceptor (redaporfin) and on the exchange rate of αβαβ could not be characterized at 37 °C because it was to fast to be characterized using a conventional fluorimeter and the stopped-flow equipment used is not sensitive at the wavelengths of redaporfin fluorescence emission. The results obtained for exchange at 25 °C are shown in Figure S6.

The exchange rate constant is essentially independent on the acceptor-to-donor LUVs ratio and very similar values are obtained when following the increase in the fluorescence from the FRET donor or the decrease in the fluorescence from the FRET acceptor. The increase in the fluorescence variation with the acceptor-to-donor LUVs ratio is also well described assuming the same affinity of αβαβ for the donor and acceptor LUVs. The maximum variation of fluorescence intensities predicted at complete transfer of αβαβ to the blank LUVs were very similar to those obtained for the transfer of α_4.

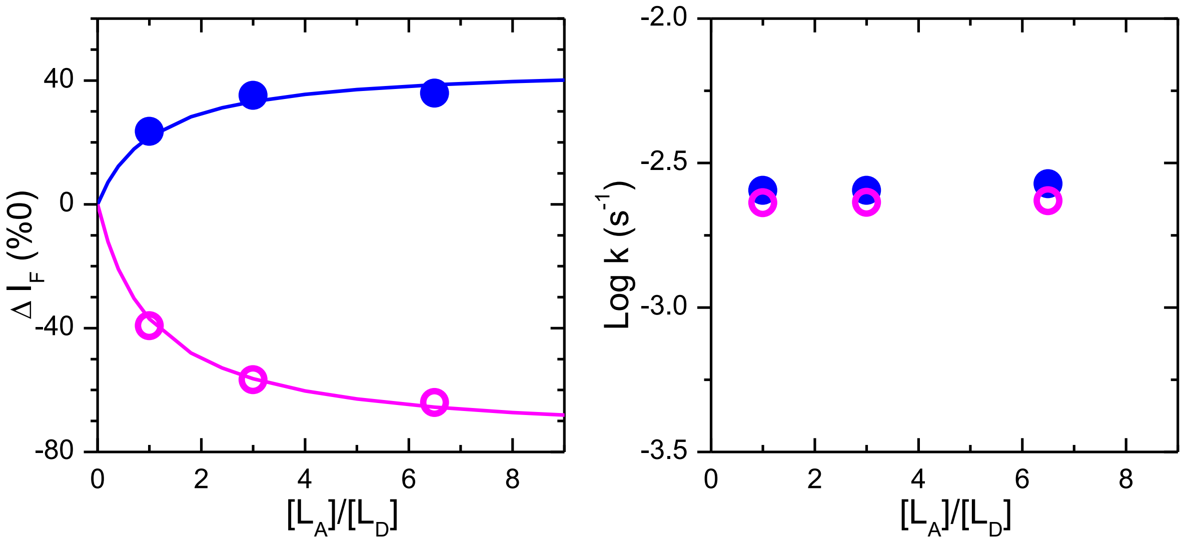

Figure S16 – Dependence of the magnitude of fluorescence variation (left plot) and average exchange rate constant (right plot) on the ratio of acceptor-to-donor LUVs, for the exchange of αβαβ between POPC:POPDE 4:1 LUVs at 25 °C. The results obtained from the variation of NBD-DPPE fluorescence (λexc=460/530 nm) are shown by filled circles in blue, and those from the variation of αβαβ fluorescence (λexc=460/752 nm) are shown as open circles in magenta. The lines in the left plot correspond to the best fit of equations (S3) to (S5).

Section S8 – Liposome size and polydispersity.

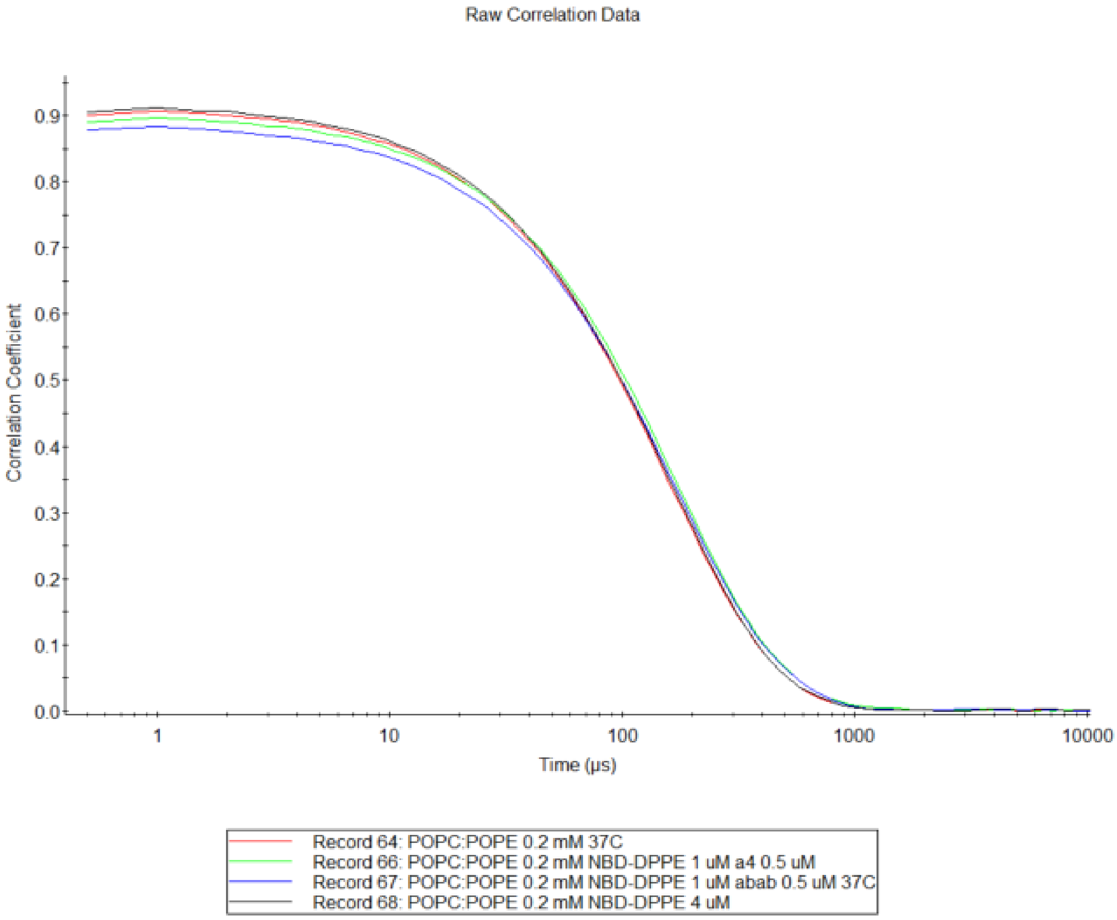

			Size (d.nm):	% Intensity:	St Dev (d.nm):
Z-Average (d.nm):	109.2	Peak 1:	126.4	100.0	51.22
PdI:	0.126	Peak 2:	0.000	0.0	0.000
Intercept:	0.955	Peak 3:	0.000	0.0	0.000
Result quality :	Good				

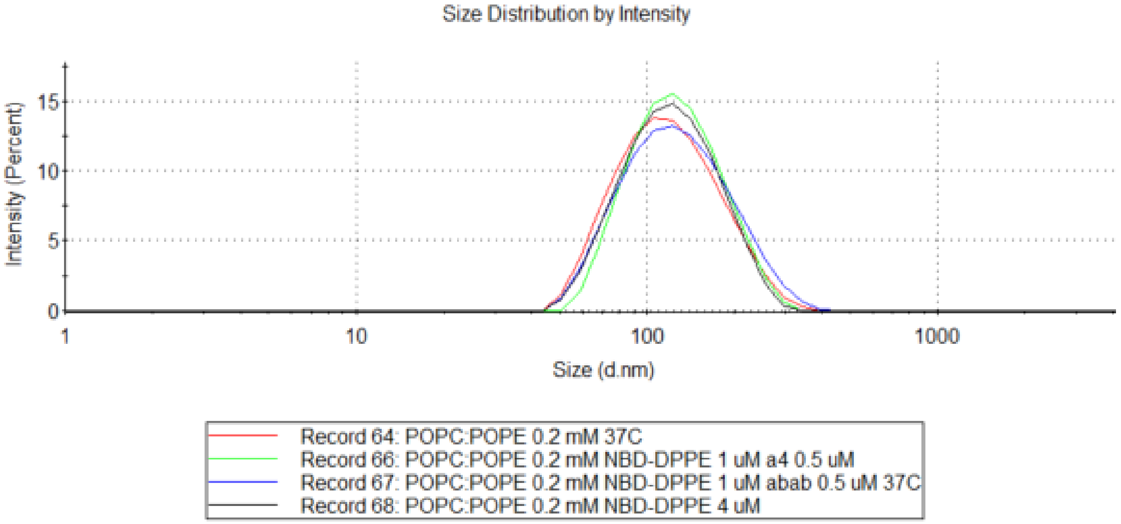

			Size (d.nm):	% Intensity:	St Dev (d.nm):
Z-Average (d.nm):	118.7	Peak 1:	132.1	100.0	46.80
PdI:	0.127	Peak 2:	0.000	0.0	0.000
Intercept:	0.949	Peak 3:	0.000	0.0	0.000
Result quality :	Good				

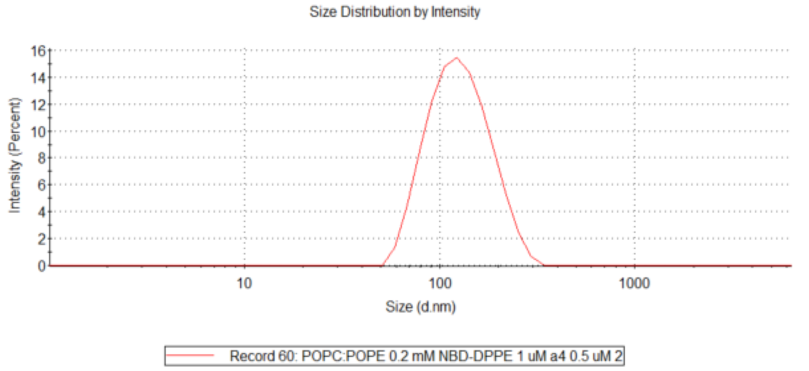

			Size (d.nm):	% Intensity:	St Dev (d.nm):
Z-Average (d.nm):	116.4	Peak 1:	135.4	100.0	56.97
PdI:	0.148	Peak 2:	0.000	0.0	0.000
Intercept:	0.941	Peak 3:	0.000	0.0	0.000
Result quality :	Good				

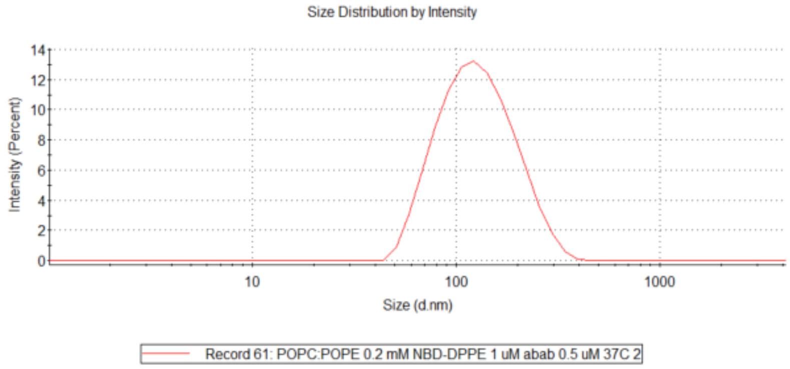

			Size (d.nm):	% Intensity:	St Dev (d.nm):
Z-Average (d.nm):	110.0	Peak 1:	127.2	100.0	46.44
PdI:	0.127	Peak 2:	0.000	0.0	0.000
Intercept:	0.957	Peak 3:	0.000	0.0	0.000
Result quality :	Good				

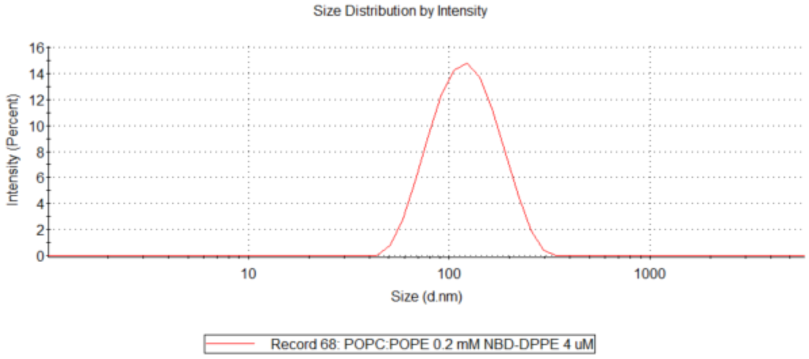

Figure S17 – Size distribution of the different LUVs used in the exchange experiments, including blank LUVs, LUVs containing NBD-DPPE and redaporfin, and LUVs containing only NBD-DPPE. All LUV samples were monodisperse, with an average diameter slightly higher than 100 nm. The size and polydispersity of the blank LUVs and those containing only NBD-DPPE was very similar (z-Average 109 nm *vs* 110 nm, and PdI 0.126 *vs* 0.127). The average size of the LUVs containing also redaporfirin was somewhat larger (z-Average 132 nm and 135 nm, for α_4 and αβαβ respectively) and the LUVs containing αβαβ showed a larger polidispersity (PdI=0.148).

Section S9 – Redaporfin reduction by dithionite.

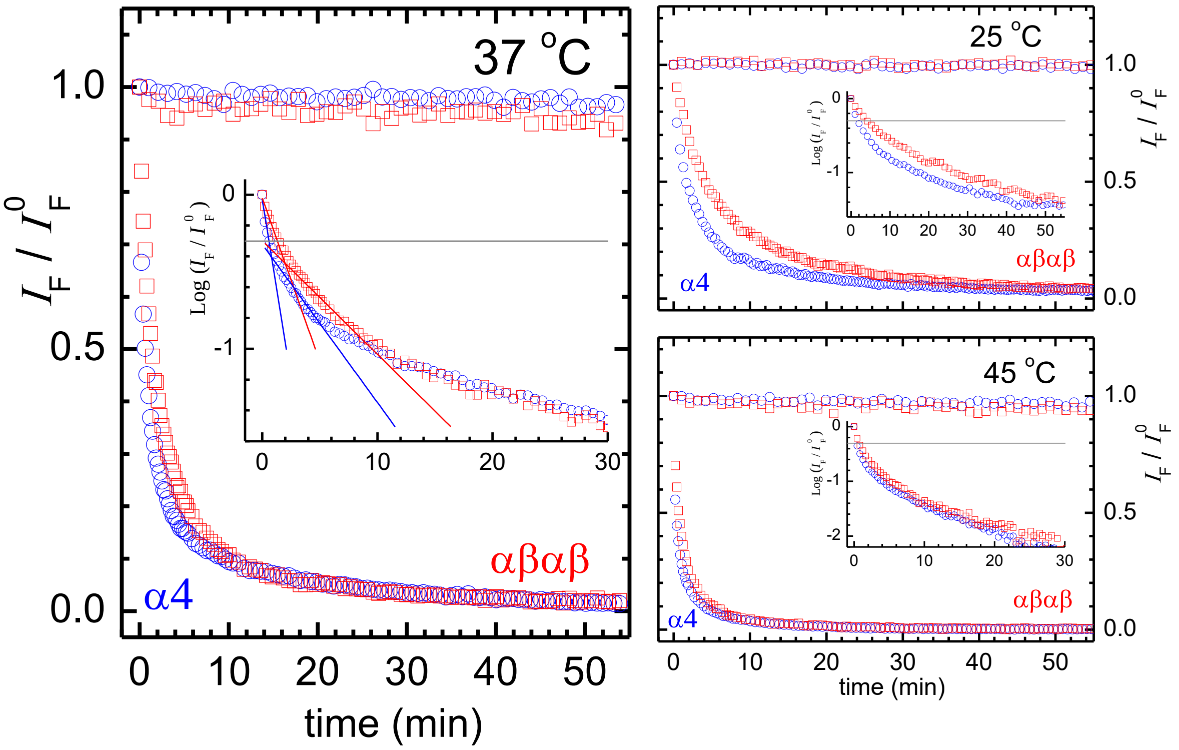

Figure S18 – Decrease in redaporfin fluorescence intensity due to reduction by dithionite (10 mM). The inserts show the fluorescence variation in a logarithmic scale, highlighting the multiexponential behavior. The horizontal grey line in the inserts corresponds to $I_F/I_F^0 = 0.5$, and the colored lines in the insert at 37 °C correspond to the linear best fit of $\log(I_F/I_F^0)$ for the variation of I_F/I_F^0 from 1 to 0.5 and from 0.5 to 0.1.

When 10 mM dithionite is added to POPC:POPE 4:1 LUVs containing 0.5 mol% redaporfin in degassed solutions, the fluorescence of redaporfin continuously decreases and vanishes in less than 1 hour at 37 °C. The decrease in the fluorescence intensity is multi-exponential, as highlighted by the non-linear behavior in the log scale shown in the inserts. The rate of fluorescence decrease increases with the increase in temperature, with I_F/I_F^0 for α_4 decreasing to about 0.5 within 100 s at 25 °C, 40 s at 37 °C, and 20 s at 45 °C. The fluorescence decrease is somewhat slower for αβαβ, with I_F/I_F^0 decreasing to about 0.5 within 250 s at 25 °C, 80 s at 37 °C, and 40 s at 45 °C.

The multi-exponential behavior suggests a relatively fast reaction of dithionite with redaporfin initially in the outer leaflet of the LUVs, followed by a slower reaction with redaporfin initially in the inner leaflet. This slower step could be due to translocation of redaporfin to the outer leaflet or to permeation of dithionite into the aqueous medium inside the LUVs. Dithionite permeation through POPC LUVs has been previously characterized and is a very slow process that occurs in the time scale of several hours [7]. This suggests that the decrease in $\frac{I_F}{I_F^0}$ below 0.5 could be reporting on redaporfin translocation from the inner to the outer leaflet. The rate of this step is similar for both redaporfin atropisomers, being around 10^{-3} s^{-1} at 25 °C, 3×10^{-3} s^{-1} at 37 °C, and 4×10^{-3} s^{-1} at 45 °C. Care should however be given to a quantitative interpretation because the rate of this step depends on the rate of redaporfin translocation, dithionite permeation, and redaporfin reaction with dithionite. Nevertheless, the results strongly suggest that the rate of α_4 translocation is much faster than the rate of exchange between LUVs, indicating that desorption from the membrane is the rate limiting step for this redaporfin atropisomer. In contrast, the rate of αβαβ fluorescence decrease due to reaction with dithionite occurs on a similar time scale to the exchange between LUVs suggesting that translocation and desorption occur at similar rates.

Section S10 – Evaluation of NBD-DPPE and redaporfin purity.

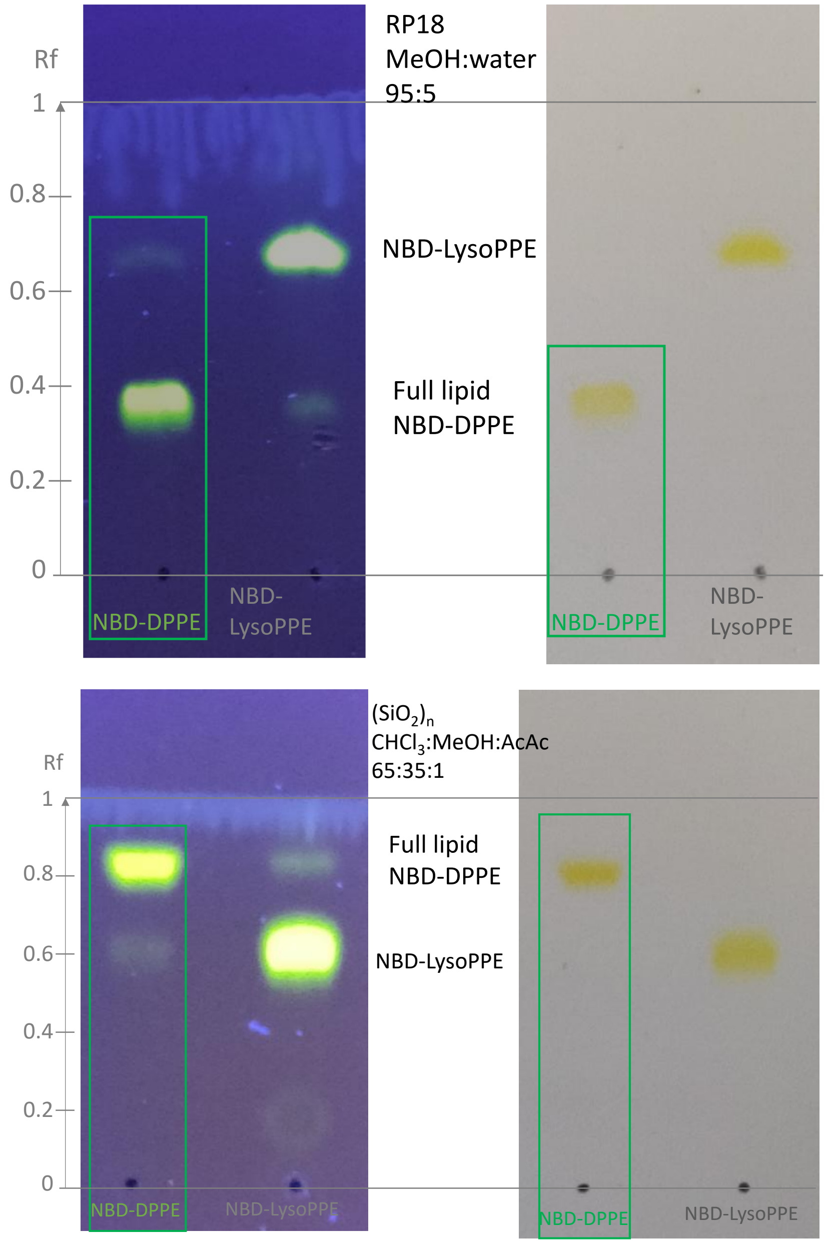

Figure S19 – Evaluation of NBD-DPPE purity. The purity of the sample used in this work was evaluated by TLC using RP18 plates with MeOH:H2O 95:5 as eluent (top lots) and by regular silica plates with CHCl3:MeOH:AceticAcid 65:35:1 (bottom plots). The plates with visualized by fluorescence with excitation at 336 nm (left plots), and under visible light (right plots). The elution profile of NBD-DPPE is compared with that of the most probable hydrolysis product (NBD-LysoPPE) to evaluate for the presence of impurities and/or degradation products.

The results obtained by TLC, using both reverse phase (top plots) and normal (bottom plots) plates, show that the NBD-DPPE sample used in this work is essentially free from hydrolysis products. The full lipid, with two acyl chains, displays a high affinity for the RP18 plate (Rf ≅ 0.35) while the hydrolysis product with only one acyl chain interacts strongly with the mobile phase (Rf ≅ 0.7). When the plates are visualized under visible ambient light (right plots), a single band is observed in each sample showing that it is essentially pure. However, visualization under excitation at 366 nm shows that very small amounts of NBD-lysoPPE are visible in the NDP-DMPE sample, and vice versa. A quantification of the two components was obtained by HPLC (RP18 column with MeOH:water 95:5 as eluent) and showed that NDP-DMPE corresponds to more than 98% of the absorption at 460 nm and of the fluorescence for λex/λem=460/530 nm.

The results obtained with the regular silica plates (bottom panels), confirm the purity of the NBD-DPPE sample. As expected, the retention factors are reversed when compared with the RP18 plates, with the major band of the NBD-DPPE sample showing a lower affinity for the plate (Rf $\cong$ 0.8) than the more polar hydrolysis product NBD-LysoPPE (Rf $\cong$ 0.6).

Redaporfin atropisomers were separated and purified as previously described [5].

Section S11 – Selected permeation events from the TTMetaD simulations.

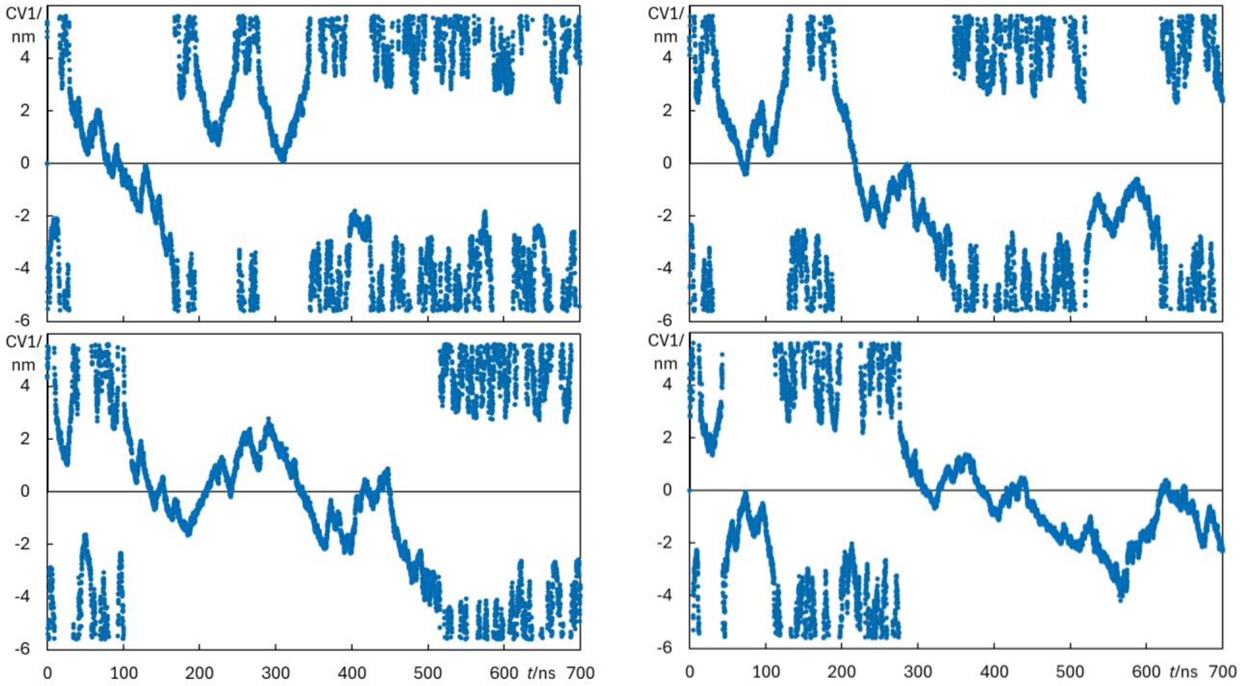

Figure S20 –Variation of the CV1 coordinate for all TTMetaD simulations of the α_4 atropisomer of redaporfin.

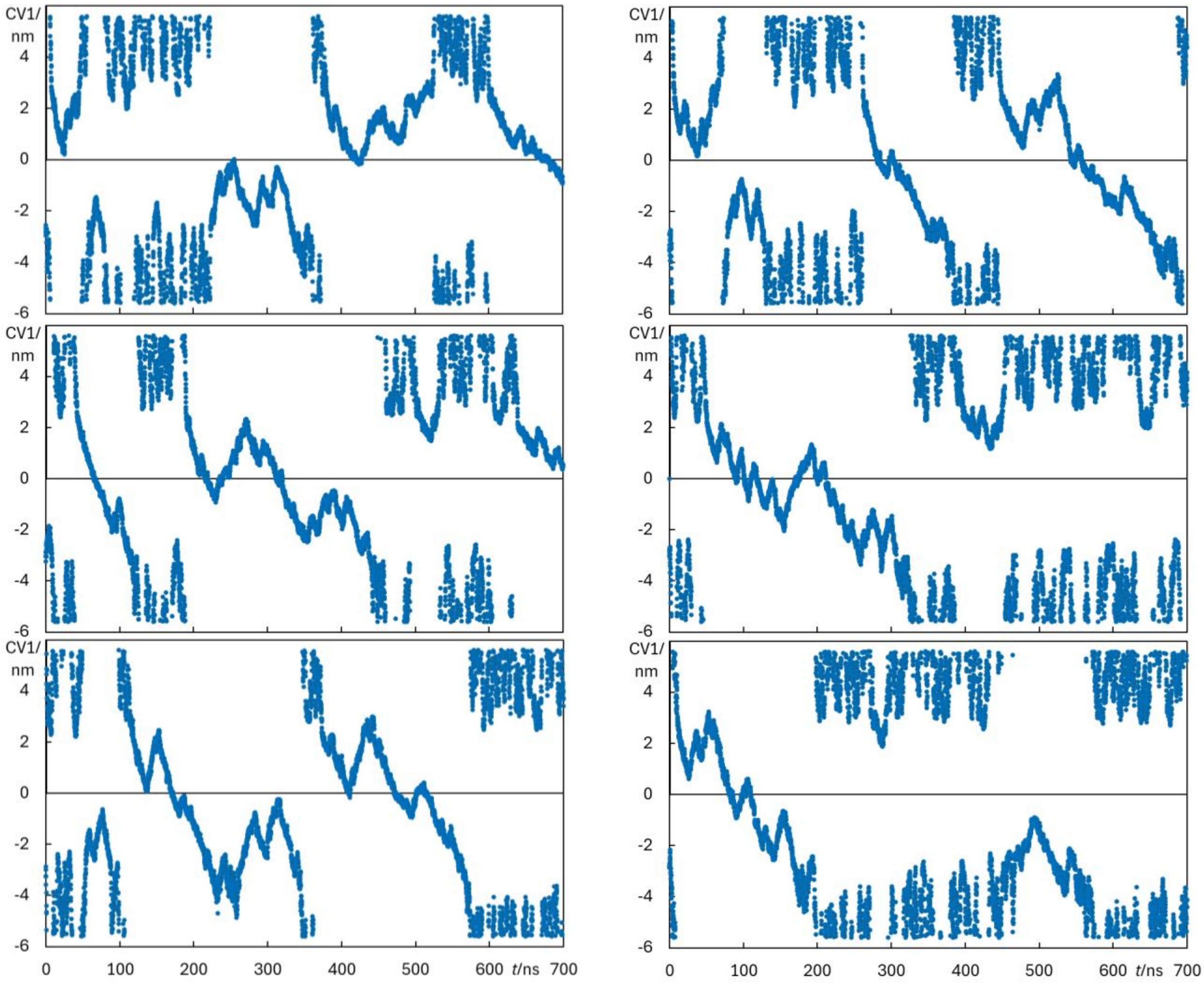

Figure S21 –Variation of the CV1 coordinate for all TTMetaD simulations of the $\alpha\beta\alpha\beta$ atropisomer of redaporfin.

α_4

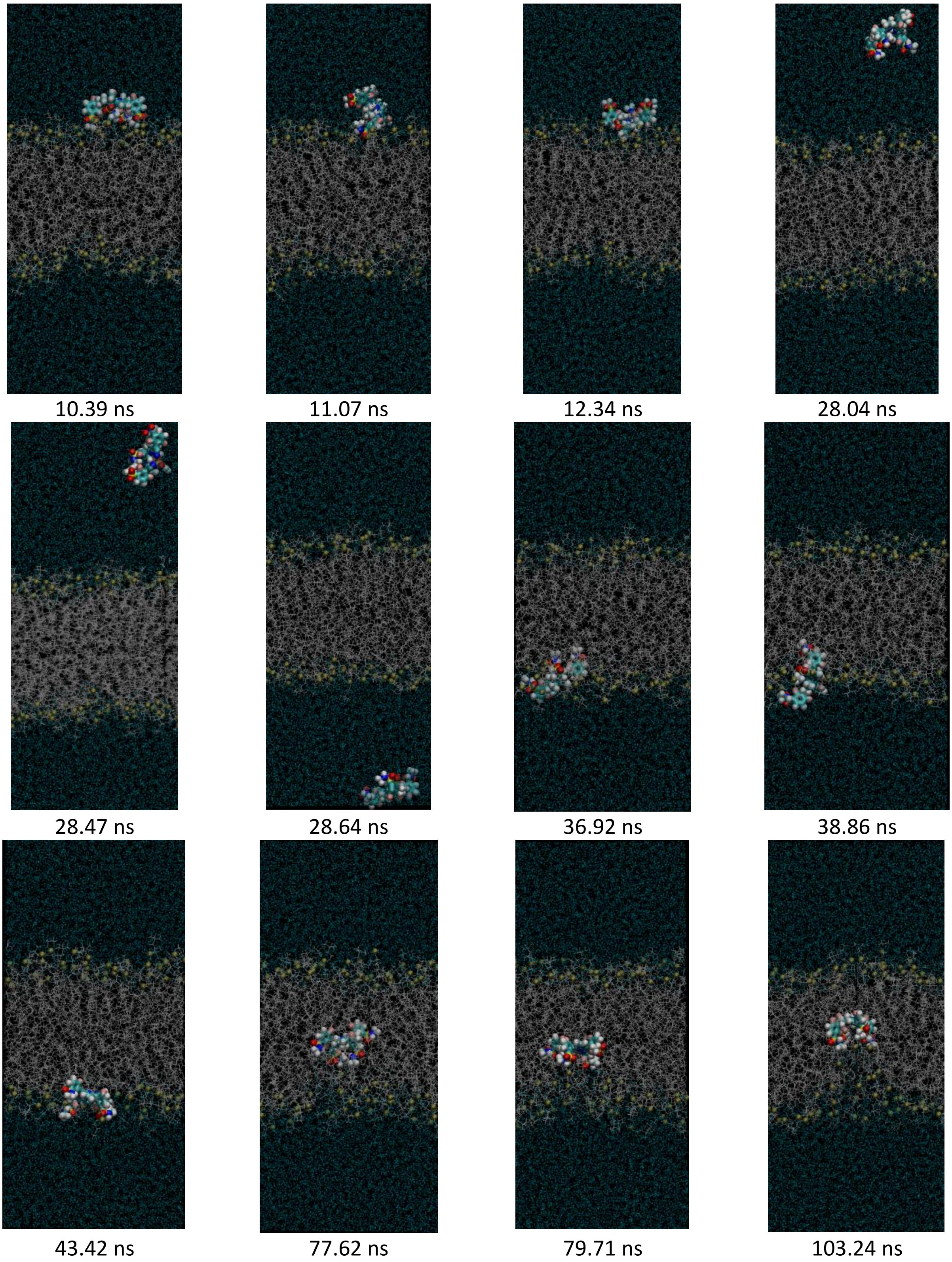
10.39 ns
11.07 ns
12.34 ns
28.04 ns
28.47 ns
28.64 ns
36.92 ns
38.86 ns
43.42 ns
77.62 ns
79.71 ns
103.24 ns

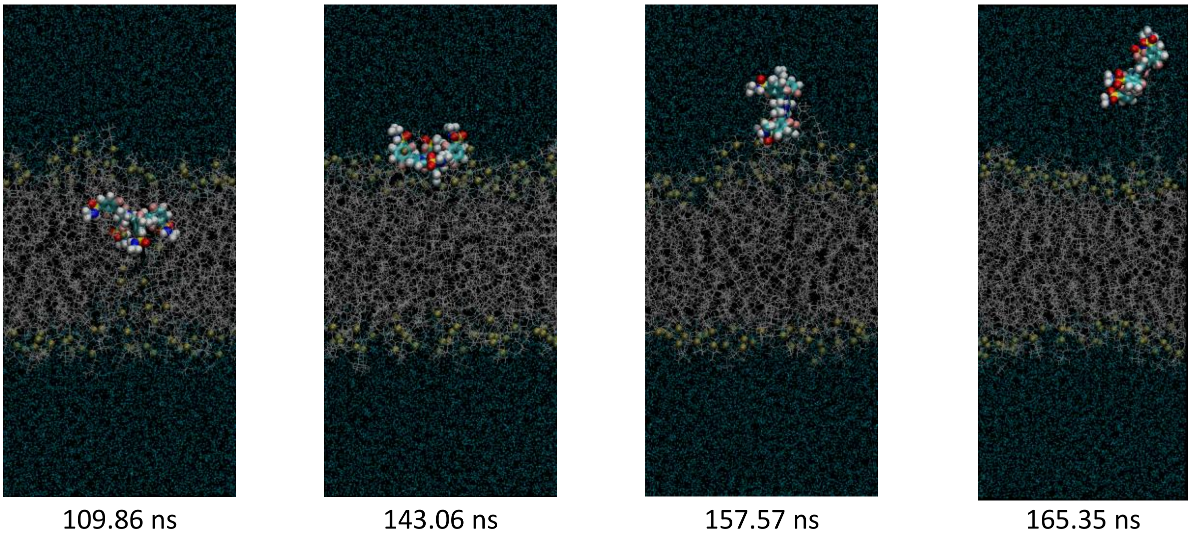

Figure S22 – Snapshots illustrating a full permeation event observed in one of the TTMetaD simulations of the α_4 atropisomer of redaporfin. The simulated molecule adsorbs at the surface, undergoes rotation while adsorbed, but does not insert (10-12 ns). It then desorbs to the aqueous medium, crosses the simulation box to the opposite side, and adsorbs again, with the sulfanamide moieties pointing to the lipid headgroups. Following this, it inserts while undergoing rotation to internalize the bacteriochlorin group and orient the sulfonamides towards the lipid head groups (37-43 ns). From this point to ~110 ns, the molecule steadily translocates, reorienting when it reaches the upper leaflet between 100 and 125 ns, to present the sulfonamide groups to the interface and keep the macrocycle in a relatively deep location (120-150 ns). Finally, the solute rotates and desorbs into the water phase, between 150 and 160 ns.

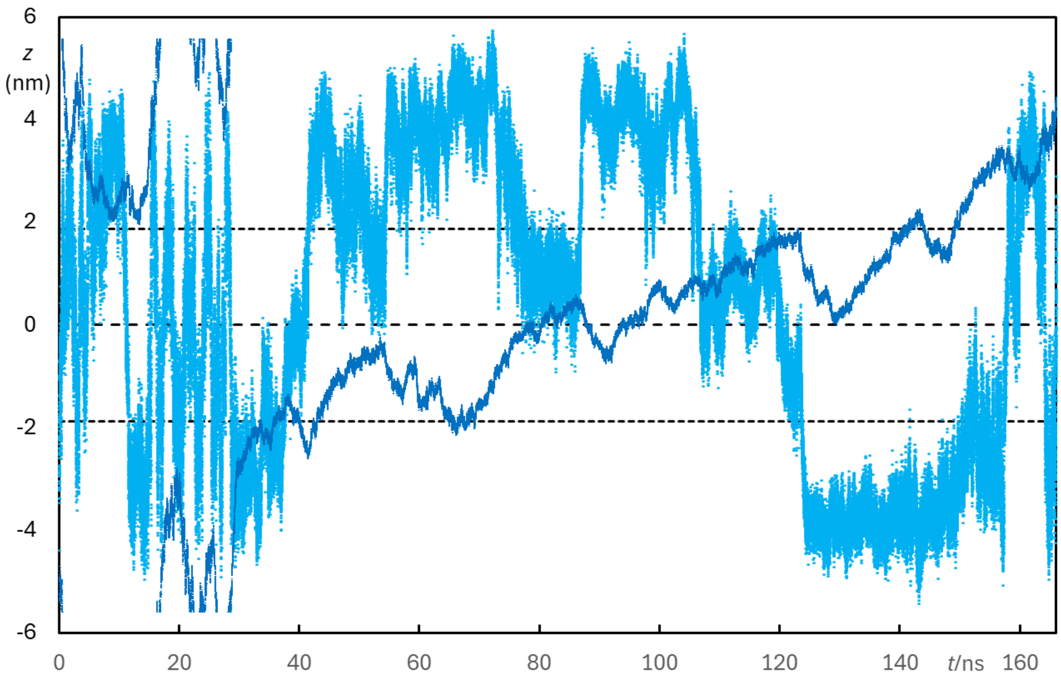

Figure S23 – Variation of CV1 (dark blue) and 10x(CV1-CV2) (light blue) for the simulation and time range illustrated in Figure S22. The dotted lines correspond to the average location of the lipid phosphorous atoms in the two leaflets.

αβαβ

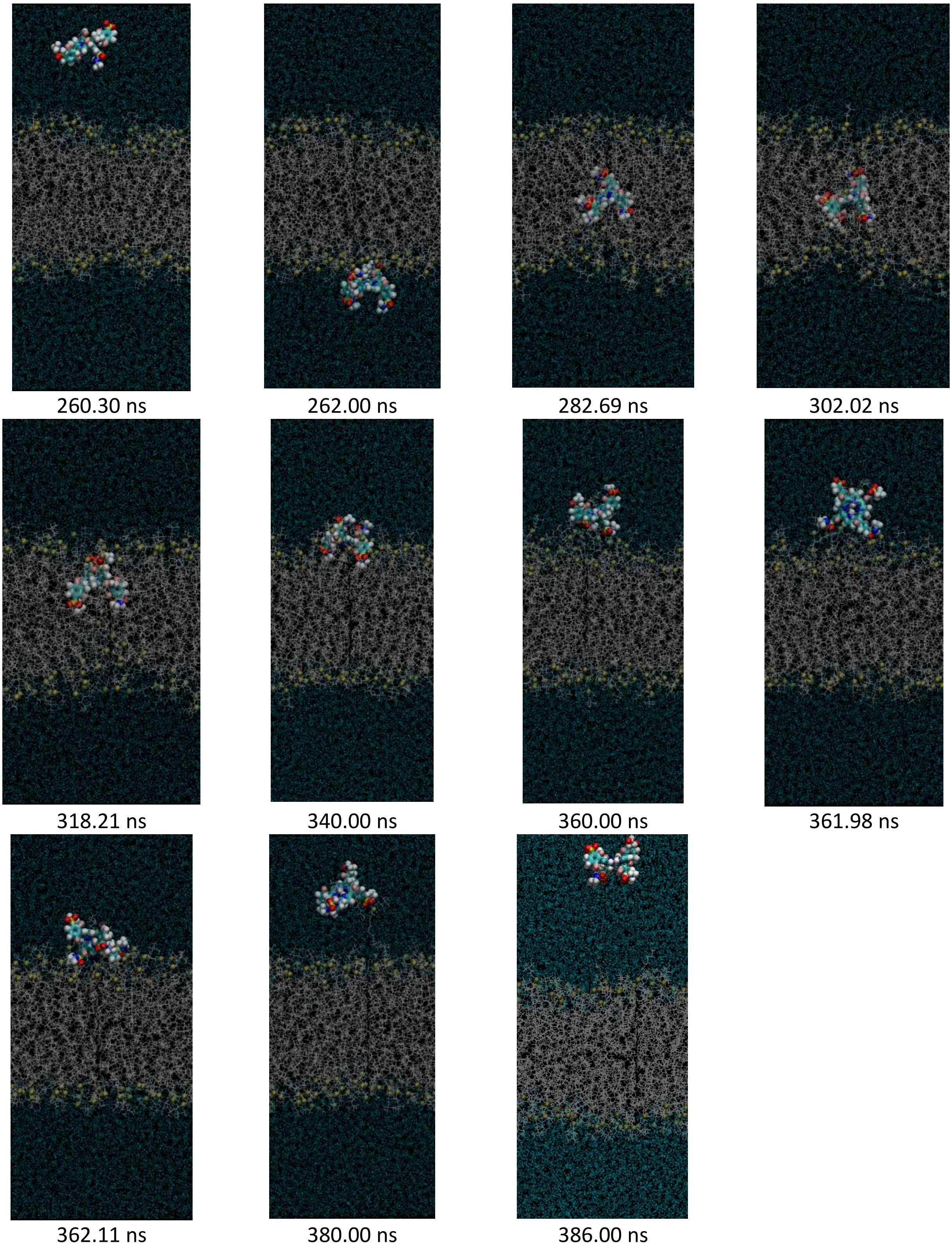

Figure S24 – Snapshots illustrating a full permeation event observed in one of the TTMetaD simulations of the αβαβ atropisomer of redaporfin. The simulated molecule adsorbs at the surface at 262 ns, and translocates steadily without rotation, until 320 ns. Afterwards, it moves to a superficial location, rotates and desorbs in the 340-380 ns time range.

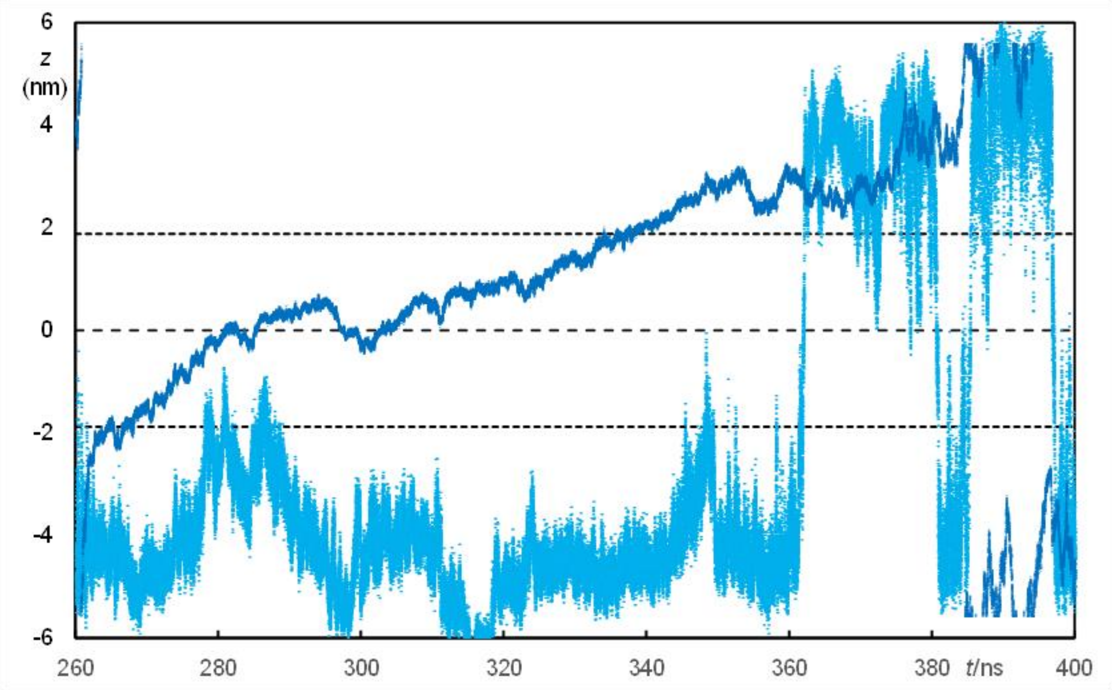

Figure S25 – Variation of CV1 (dark blue) and 10x(CV1-CV2) (light blue) for the simulation and time range illustrated in Figure S24. The dotted lines correspond to the average location of the lipid phosphorous atoms in the two leaflets.

αβαβ

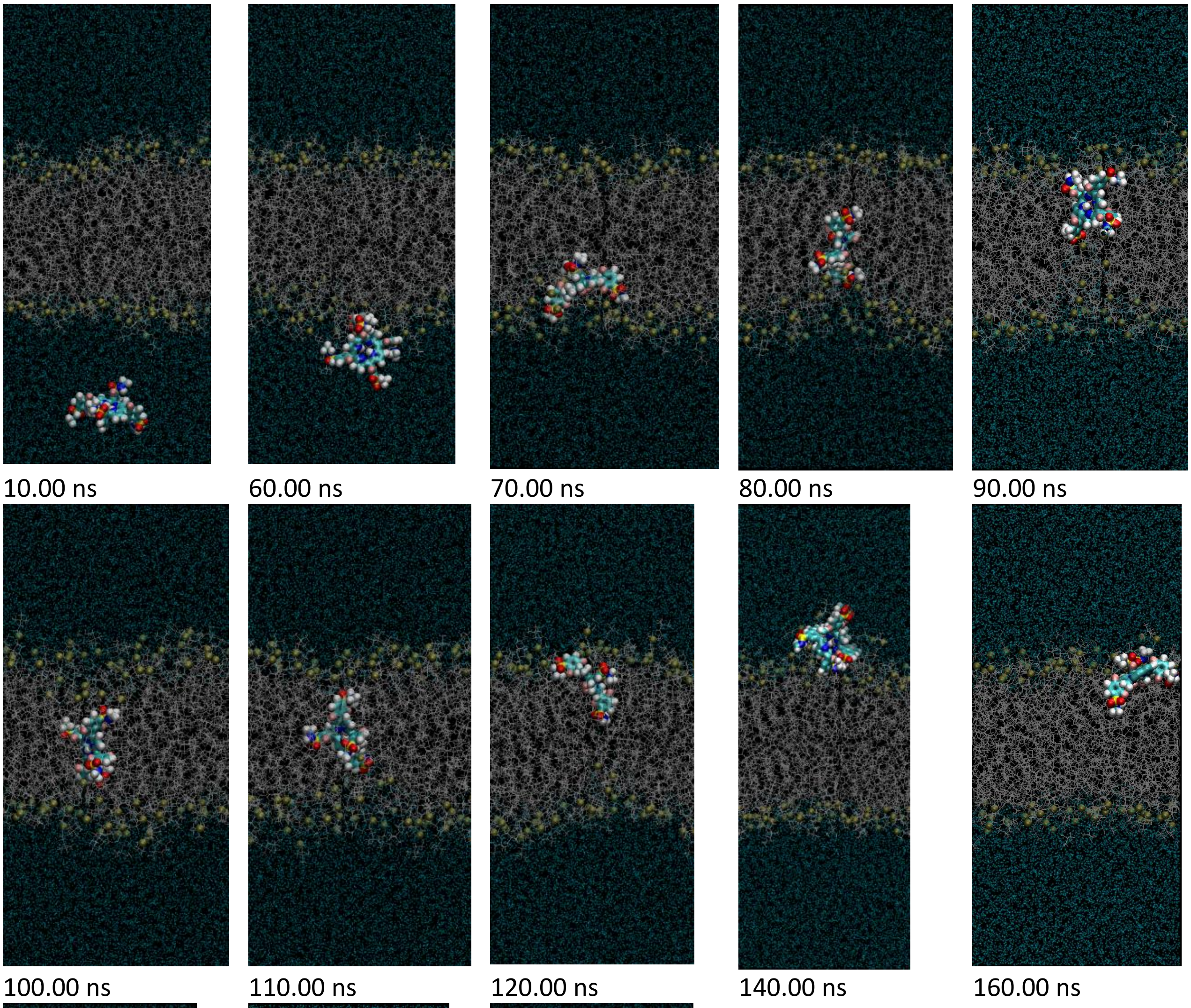

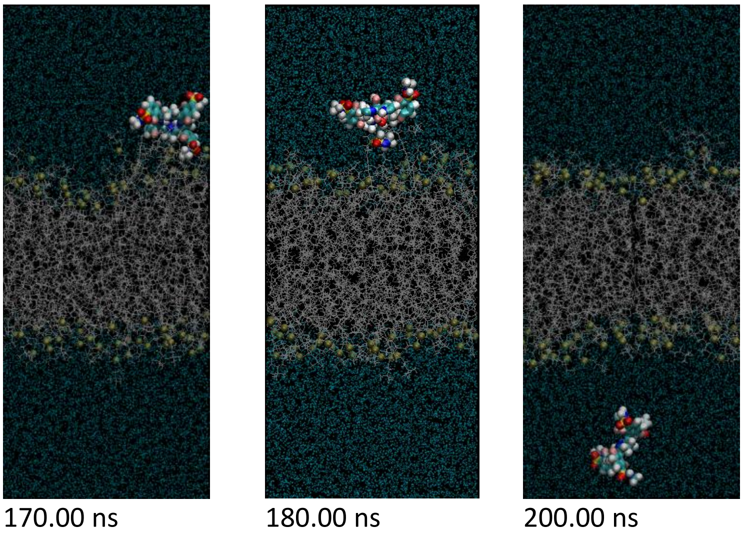

Figure S26 – Snapshots illustrating a full permeation event observed in one of the TTMetaD simulations of the αβαβ atropisomer of redaporfin (distinct from that of Figures S23-S24). The simulated molecule inserts at 60-70 ns, translocates with the bacteriochlorin ring aligned with the lipid acyl chains (80 ns), returns to the center (110 ns) and again to the upper leaflet (120 ns). It then relocates to the surface, undergoing reorientation, and finally desorbs at 170 ns, emerging at the bottom of the simulation box at 200 ns.

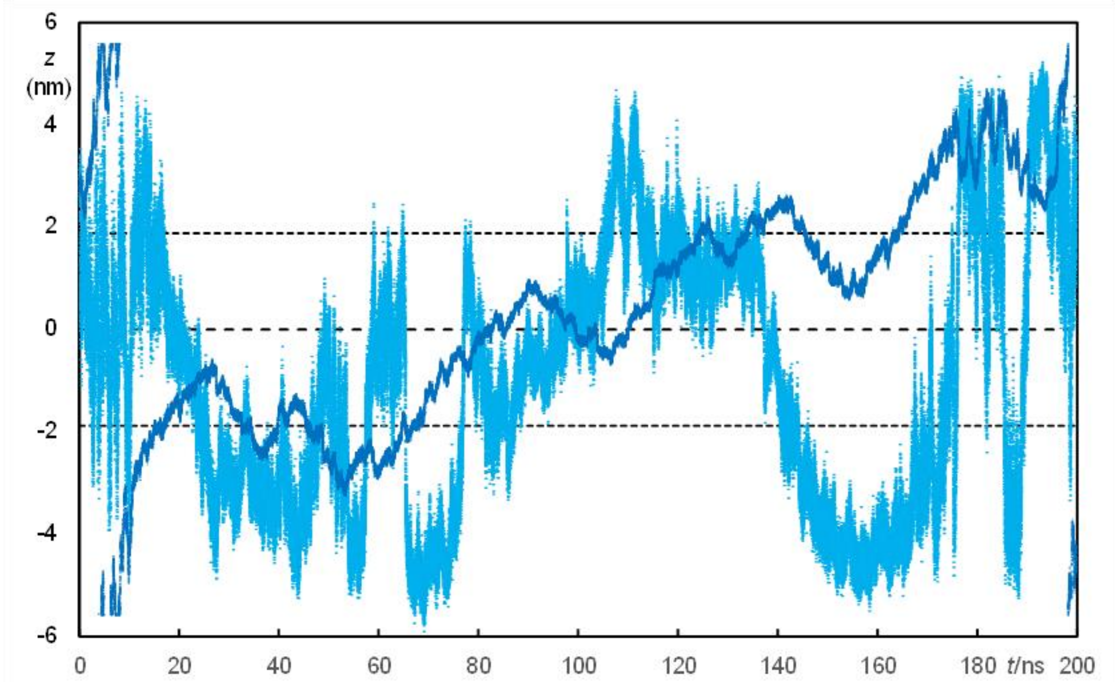

Figure S27 – Variation of CV1 (dark blue) and 10x(CV1-CV2) (light blue) for the simulation and time range illustrated in Figure S26. The dotted lines correspond to the average location of the lipid phosphorous atoms in the two leaflets.

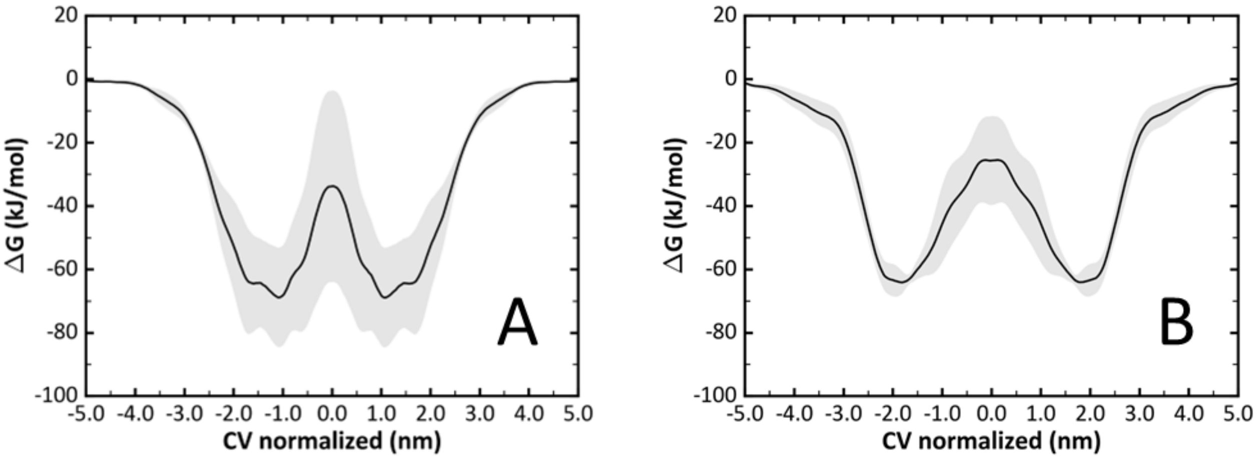

Figure S28 – One-dimensional free energy profiles of α_4 (A) or αβαβ (B) calculated along the minimum free energy path, and projected onto the CV1 coordinate. Uncertainty estimates (standard error) are shown in grey.

References

1. Cardoso, R. M. S.; Martins, P. A. T., et al., Effect of dipole moment on amphiphile solubility and partition into liquid ordered and liquid disordered phases in lipid bilayers. *BBA-Biomemb.* **2020,** *1862* (3), 183157. DOI:10.1016/j.bbamem.2019.183157
2. Ding, W.; Palaiokostas, M., et al., Effects of Lipid Composition on Bilayer Membranes Quantified by All-Atom Molecular Dynamics. *J. Phys. Chem. B* **2015,** *119* (49), 15263-15274. DOI:10.1021/acs.jpcb.5b06604
3. Brockman, H., Dipole Potential of Lipid-Membranes. *Chem. Phys. Lipids* **1994,** *73* (1-2), 57-79.
4. Bani-Yaseen, A. D.; Hammad, F., et al., On the Photophysicochemical Properties of Selected Fluoroquinolones: Solvatochromic and Fluorescence Spectroscopy Study. *J. Fluoresc.* **2013,** *23* (1), 93-101. DOI:10.1007/s10895-012-1120-7
5. Donohoe, C.; Schaberle, F. A., et al., Unraveling the Pivotal Role of Atropisomerism for Cellular Internalization. *JACS* **2022,** *144* (33), 15252-15265. DOI:10.1021/jacs.2c05844
6. Chattopadhyay, A., Chemistry and Biology of N-(7-Nitrobenz-2-Oxa-1,3-Diazol-4-Yl)-Labeled Lipids - Fluorescent-Probes of Biological and Model Membranes. *Chem. Phys. Lipids* **1990,** *53* (1), 1-15.
7. Moreno, M. J.; Estronca, L. M. B. B., et al., Translocation of phospholipids and dithionite permeability in liquid-ordered and liquid-disordered membranes. *Biophys. J.* **2006,** *91* (3), 873-881. DOI:10.1529/biophysj.106.082115
